# Synergic Neuroprotection Between *Ligusticum Chuanxiong* Hort and Borneol Against Ischemic Stroke by Neurogenesis *via* Modulating Reactive Astrogliosis and Maintaining the Blood–Brain Barrier

**DOI:** 10.3389/fphar.2021.666790

**Published:** 2021-06-16

**Authors:** Bin Yu, Yao Yao, Xiaofeng Zhang, Ming Ruan, Zhennian Zhang, Li Xu, Tao Liang, Jinfu Lu

**Affiliations:** ^1^ Jiangsu Key Laboratory for Pharmacology and Safety Evaluation of Chinese Materia Medica, Nanjing University of Chinese Medicine, Nanjing, China; ^2^ School of Food Science, Nanjing Xiaozhuang University, Nanjing, China; ^3^ Department of Encephalopathy, Nanjing Hospital of Traditional Chinese Medicine, Nanjing, China

**Keywords:** *Ligusticum chuanxiong* Hort., borneol, ischemic stroke, neurogenesis, blood–brain barrier

## Abstract

**Background:** *Ligusticum chuanxiong* Hort (LCH) is a famous ethnomedicine in Asia known for its excellent output on stroke treatment, and borneol usually acts as an assistant for its reducing permeability of the blood–brain barrier (BBB) after stroke. Although their synergy against brain ischemia was verified in previous studies, the potential mechanism is still unknown.

**Methods:** The research aimed to explore the exact synergic mechanisms between LCH and borneol on neurogenesis within the areas of the dentate gyrus and subventricular zone. After treating middle cerebral artery occlusion rats with LCH (0.1 g/kg) and/or borneol (0.08 g/kg), the neurological severity score, brain infarct ratio, Nissl staining, Evans blue permeability, BBB ultrastructure, and expressions of von Willebrand factor and tight junction–associated proteins were measured. Co-localizations of Nestin^+^/BrdU^+^ and doublecortin^+^/BrdU^+^, and expressions of neuronal nuclei (NeuN) and glial fibrillary acidic protein (GFAP) were observed under a fluorescence microscope. Moreover, astrocyte polarization markers of complement component 3 and pentraxin 3, and relevant neurotrophins were also detected by immunoblotting.

**Results:** Basically, LCH and borneol had different focuses, although both of them decreased infarct areas, and increased quantity of Nissl bodies and expression of brain-derived neurotrophic factor. LCH increased the neurological severity score, NeuN^+^ cells, and the ratios of Nestin^+^/BrdU^+^ and doublecortin^+^/BrdU^+^, and decreased GFAP^+^ cells and ciliary neurotrophic factor expression. Additionally, it regulated the expressions of complement component 3 and pentraxin 3 to transform astrocyte phenotypes. Borneol improved BBB ultrastructure and increased the expressions of von Willebrand factor, tight junction–associated proteins, vascular endothelial growth factor, and vascular endothelial growth factor receptor 2. Unexpectedly, their combined therapy showed more obvious regulations on the Nissl score, Evans blue permeability, doublecortin^+^/BrdU^+^, NeuN^+^ cells, brain-derived neurotrophic factor, and vascular endothelial growth factor than both of their monotherapies.

**Conclusions:** The results indicated that LCH and borneol were complementary to each other in attenuating brain ischemia by and large. LCH mainly promoted neural stem cell proliferation, neurogenesis, and mature neuron preservation, which was probably related to the transformation of reactive astrocytes from A1 subtype to A2, while borneol preferred to maintain the integrity of the BBB, which provided neurogenesis with a homeostatic environment.

## Introduction

Stroke, as a leading cause of death and disability, attacks about 15 million peoples worldwide each year, and ischemic stroke, caused by deficiency of cerebral blood flow, makes up about 80–85% of all strokes (Alwjwaj et al., 2021). Except antithrombotic therapy, there is no ideal therapy in clinic. However, the antithrombotic drugs, as represented by recombinant tissue plasminogen activator (rtPA), only provided a transient therapy window within 4.5 h (Lansberg et al., 2009), and even usually produces a wide range of side effects, such as bleeding, dizziness, and headache (Liu et al., 2018). So, discovering novel, highly effective, and low toxicity anti-ischemic stroke drugs with clear mechanism is one of the hot topics in pharmacologic research.

Neurogenesis, developed by endogenous neural stem cells (NSCs) with the ability to replace damaged neurons, is regarded as a potential therapeutic strategy of brain ischemia (Lindvall and Kokaia, 2011; Abe et al., 2012). Additionally, it has been demonstrated that NSCs are restricted within the subventricular zone (SVZ) of the lateral ventricles and the subgranular zone of the dentate gyrus (DG) in the normal adult brains (Navarro Negredo et al., 2020; Ottoboni et al., 2020), and is activated by ischemic stress (Hou et al., 2017). Nestin, DCX, and GFAP are usually regarded as biomarkers of neural stem cells, newborn neurons, and astrocytes, respectively (Chen et al., 2020; Yin et al., 2020). Additionally, a growing number of reports suggest that an array of neurotrophic factors are synthesized and secreted into the neurogenic microenvironment to address ischemic stroke (Wu, 2005; Hedayatpour et al., 2018; Zalewska et al., 2020). The identified factors and receptors include brain-derived neurotrophic factor (BDNF), tyrosine kinase receptor B (TrkB) (Jiang et al., 2021), nerve growth factor (NGF) (Zalewska et al., 2020), basic fibroblast growth factor (bFGF), vascular endothelial growth factor (VEGF), vascular endothelial growth factor receptor 2 (VEGFR2) (Fei et al., 2018), ciliary neurotrophic factor (CNTF) (Jia et al., 2018), and neurotrophin-3 (NT-3) (Cruz et al., 2015). These neurotrophins produce different regulations on proliferation of NSCs, regeneration of neurons and astrocytes (ACs), and even maintenance of the blood–brain barrier (BBB) *via* a complex network in postischemic brain.

It is reported that there are two phenotypes of ACs, known as A1 and A2, in reactive astrocytosis. A2 ACs, marked with pentraxin 3 (PTX3), participate in the developments of neurons and synapses *via* releasing various neurotrophins, while A1 ACs, marked with complement component 3 (C3), is considered to be harmful because of its ability to kill central nervous system (CNS) neurons (Zamanian et al., 2012). Moreover, it is demonstrated that both long-term hypoperfusion and ischemia induce the increase of A1-type ACs, and even form a glial scar (Miyamoto et al., 2020). Thus, the transformation of AC phenotypes from A1 to A2 is regarded as a potential treatment strategy for ischemic stroke.

According to the theory of traditional Chinese Medicine, stroke is caused by blood stasis, and its treatment mainly depends on stasis-eliminating therapy. *Ligusticum chuanxiong* Hort (LCH) is one of the most common Chinese herbal medicines possessing stasis-eliminating function (Li et al., 2015; Chen et al., 2018). LCH injection is its commercial product and widely used for ischemic stroke patients in Asia. Previous studies suggest that LCH reduces cerebral infarct and inflammatory reaction, improves neurological behavior (Ip et al., 2016), and alleviates oxidative stress and neuronal apoptosis of middle cerebral artery occlusion (MCAO) rats (Gu et al., 2020). Borneol (BO), another Chinese ethnomedicine with a bicyclic terpene structure, is extracted from *Blumea balsamifera* (L.) DC. or *Cinnamomum camphora* (L.) Presl. There are numerous evidences indicating that BO significantly reduces brain water content, alleviates brain edema, and maintains the BBB in cerebral ischemic injury (Zhang X.-g. et al., 2017; Chen et al., 2019). Additionally, BO is more often used as an assistant in CNS treatment, especially for stroke, to produce a synergic therapeutic effect basing on traditional Chinese Medicine theory (Zhang X.-G. et al., 2017; Liao et al., 2020). Our early studies have confirmed that the combination of LCH and BO exerts a much better protection against cerebral ischemia than their monotherapies *via* downregulation of apoptosis, upregulation of autophagy, and angiogenesis (Yu et al., 2020a; Yu et al., 2020b). However, their respective focuses and potential synergic mechanism are still unclear.

In consideration of abovementioned results and the close relationship among neuron autophagy, neurogenesis, and NSC differentiation (Fleming and Rubinsztein, 2020), the present study was designed to explore the synergic mechanism between LCH and BO against stroke based on NSC differentiation, neurogenesis, and BBB maintenance.

## Materials and Methods

### Materials

LCH injection, a steam distillation product of LCH, was provided by Changhai Hospital of Shanghai. A total of 59 compounds were identified by ultra-performance liquid chromatography–tandem mass spectrometry (UPLC–MS/MS) technology, and 25 compounds were identified by the gas chromatography–mass spectroscopy (GC–MS) method. The detailed information is listed in Supplementary File. BO was purchased from Beijing Sanhe Pharmaceutical Co. Ltd., and its purity was 99.1%.

### Animals

Healthy male SD rats (8 weeks, 300–350 g) were purchased from the Animal Center of Nanjing University of Chinese Medicine, maintained in a light/dark cycle (12 h/12 h) room, and freely accessible to food and water. The animal protocols of the study were approved by the Animal Ethics Committee of Nanjing University of Chinese Medicine (No. 201901A007).

### Procedure For Middle Cerebral Artery Occlusion

MCAO rat is a widely used animal model of ischemic stroke because it may produce similar pathological changes to stroke patients in clinic (Wu et al., 2020; Barahimi et al., 2021), and the procedure was similar to previous reports with minor modifications (Costa et al., 2016; Ma et al., 2016). Briefly, the rat was anesthetized with 3% isoflurane in a chamber affiliated to a small animal anesthesia machine (RWD Life Science Co., LTD., China) and maintained with 1.5% isoflurane delivered through a face mask fitting the rat’s head. After the left common carotid artery was exposed, the internal and external carotid arteries were separated from each other. Then the external carotid artery was ligated by an absorbable suture. A nylon thread (diameter 0.26–0.27 mm) with its top coated with silicone (length 6–7 mm and diameter 0.41–0.45 mm) was inserted into the internal carotid artery at a depth of 18–20 mm to block the middle cerebral artery, and reperfusion was followed 1 h later. The rats in the sham group underwent the same operation, except the insertion of nylon thread. The rat’s body temperature was maintained at 37°C during the entire operation. 24 hours after the surgery, the rats with neurological severity scores (NSS) no less than three were used for the following study (Bieber et al., 2019).

### Drug Treatment and BrdU Label

Rats were randomly divided into five groups, with 19 rats in each group: sham, model, LCH, BO, and LCH + BO groups. All rats were subjected to MCAO operation, except those in the sham group. The rats in the LCH, BO, and LCH + BO groups were treated with LCH injection (i.p. 0.1 g/kg) or/and BO (i.g. 0.08 g/kg). Both the sham and the model groups were given equal volume of PBS (i.p.) and liquid paraffin (i.g.). All drugs were given once daily for 7 days, including 4 days before the MCAO surgery and 3 days after that. Additionally, the rats used for immunofluorescence analysis were injected with 5-bromo-2’-deoxyuridine (BrdU, i.p. 50 mg/kg) once daily for the last 3 days.

### Behavioral Test

After treatment for 7 days, the NSS were employed to evaluate neurological behaviors *via* a five-point Bederson scale (Bederson et al., 1986) (Bi et al., 2017). Increase of the score indicated decrease of neurological function. Specifically, 0: no abnormal behavior; 1: minor neurological deficiency (right forelimb bending when lifting its tail); 2: moderate neurological deficiency (decreased stability to left slight push) without cycling; 3: same neurological deficiency as grade 2, but cycling to right when moving; and 4: no spontaneous walking, or even unconsciousness.

### Infarct Size Measurement

2,3,5-triphenyltetrazolium chloride (TTC) staining was performed to measure the ratio of infarct size. After being anesthetized with 2% isoflurane, the rat was sacrificed by decapitation, and then its brain was taken out gently. Five brain coronal slices with a thickness of 2 mm were made by a tissue microtome. Then the slices were immersed in a 0.1% TTC PBS solution at 37°C for 15 min. Both the infarct area and the total brain area were measured using ImageJ software, and the infarction ratio was the percentage of infarct area in the total brain area.

### Score of Nissl Staining

The whole brain tissue was obtained and placed in a 4% paraformaldehyde PBS solution for 24 h. After dehydration, it was fixed in paraffin, and then cut into 4 μm coronal slices according to the stereotaxic coordinates of DG and SVZ (DG: AP −3.6 mm; SVZ: AP + 0.0 mm). Stained with 1% toluidine blue in accordance with the kit’s instruction, the intact Nissl bodies in both DG and SVZ areas were quantified as Nissl scores using a microscope (IX71, Olympus, Tokyo, Japan) under five random fields.

### Blood–Brain Barrier Permeability Evaluation

The rat was injected with 4 ml/kg of 2% Evans blue (EB) *via* caudal vein. 3 hours later, the rat was anesthetized with isoflurane and perfused with PBS *via* left ventricle to remove the intravascular EB. After pictures were taken, the content of EB in brain tissue was measured according to previous reports (Nozaki et al., 2018; Cheng et al., 2021). Briefly, the ischemic side hemisphere was separated, weighted, and homogenized in 3 ml of formamide. Then the sample was incubated at 37°C for 48 h, followed by centrifugation at 10,000 g for 20 min. The OD value of the supernatant was measured by a microplate reader (BioTek Instruments, Vermont, United States ) at the wavelength of 620 nm. The content of EB in the brain was calculated by an EB standard curve.

### Ultrastructure Examination

The brain tissue was prepared as our previous report (Yu et al., 2013b). Briefly, a small piece of DG or SVZ tissue was removed carefully from rat brain, fixed in 2% glutaraldehyde for 4 h, and osmicated for 1 h at 4°C with 1% OsO_4_ and 0.8% potassium ferricyanide. Then the brain section was dehydrated using acetone, embedded in Epon 812 epoxy resin, and prepared as an ultrathin section. Finally, the section was observed using a transmission electron microscope (H7650, Hitachi, Japan) after being stained with uranyl acetate and lead citrate.

### Immunofluorescence Histochemistry

After being anesthetized with isoflurane and perfused with PBS, the rat brain was removed and fixed in 4% paraformaldehyde for 72 h, and then sliced into 4- to 8-µm-thick coronal pieces in accordance with the stereotaxic coordinates of DG and SVZ mentioned above. Subsequently, the slice was mounted on a glass substrate, rinsed with PBS three times, immersed in 10% donkey serum for 1 h, and incubated overnight at 4°C with corresponding rabbit primary antibody below. For detecting NSC proliferation and neurogenesis, the primary antibodies were BrdU (1:100, catalog number: ab6326, Abcam, Cambridge, MA, United States ), Nestin (1:100, catalog number: ab221660, Abcam, Cambridge, MA, United States ), and Doublecortin (DCX, 1:200, catalog number: ab18723, Abcam, Cambridge, MA, United States ). For assessing the preservation of mature neurons and astrogliosis, the primary antibodies were neuronal nuclei (NeuN, 1:400, catalog number: ab177487, Abcam, Cambridge, MA, United States ) and glial fibrillary acidic protein (GFAP, 1:400, catalog number: ab7260, Abcam, Cambridge, MA, United States ). For evaluating the proliferation of brain microvascular endothelial cells (BMECs), the primary antibody was von Willebrand factor (vWF) (1:400, catalog number: ab6994, Abcam, Cambridge, MA, United States ). For observing expressions of TJ-associated proteins on the BBB, the primary antibodies were claudin-5 (1:50, catalog number: 310,145, Sigma-Aldrich, Saint Louis, MO, United States ), junctional adhesion molecule 3 (JAM-3, 1:100, catalog number: ab214194, Abcam, Cambridge, MA, United States ), occludin (1:50, catalog number: 71–1,500, Thermo Scientific, Waltham, MA, United States ), and zonula occludens-1 (ZO-1, 1:100, catalog number: ab221547, Abcam, Cambridge, MA, United States ). After the slice was incubated with second antibodies coupled with Alexa Fluor 488 (a green fluorescent dye) or Alexa Fluor 568 (a red fluorescent dye) for 1 h at room temperature, it was counterstained with 4′,6–diamidino–2–phenylindole (DAPI), and then visualized using a fluorescence microscopy (IX71, Olympus Tokyo, Japan). The fluorescence intensity was measured using ImageJ software.

### Western Blot

DG and SVZ tissues were removed carefully, and the proteins were extracted using a radio-immunoprecipitation assay (RIPA) buffer (Jiangsu KeyGEN BioTECH Co., Ltd.). Proteins with equal content were separated on 4–20% sodium dodecyl sulfate–polyacrylamide gel electrophoresis (SDS–PAGE) and transferred to 0.22 μm polyvinylidene fluoride (PVDF) membranes (Millipore). After being blocked by 5% bovine serum albumin (BSA), the membranes were incubated with the following primary antibodies overnight at 4°C: BDNF (1:5,000, catalog number: ab108319, Abcam, Cambridge, MA, United States), bFGF (1:2000, catalog number: PA5–95284, Invitrogen, Carlsbad, CA, United States), CNTF (1:5,000, catalog number: 10,796–1–AP, Proteintech, Rosemont, IL, United States), NGF (1:1,000, catalog number: MA5–32067, Invitrogen, Carlsbad, CA, United States), NT-3 (1:2000, catalog number: 18,084–1–AP, Proteintech, Rosemont, IL, United States), TrkB (1:1,000, catalog number: 13,129–1–AP, Proteintech, Rosemont, IL, United States), VEGF (1:2000, catalog number: 19,003–1–AP, Proteintech, Rosemont, IL, United States), VEGFR2 (1:1,000, catalog number: ab39256, Abcam, Cambridge, MA, United States), C3 (1:2000, catalog number: ab200999, Abcam, Cambridge, MA, United States), PTX3 (1:1,000, catalog number: ab134920, Abcam, Cambridge, MA, United States), and glyceraldehyde-3-phosphate dehydrogenase (GAPDH, 1:10,000, catalog number: 10,494–1–AP, Proteintech, Rosemont, IL, United States). After being washed by tris-buffered saline tween (TBST), the membranes were incubated with corresponding secondary antibodies for 1 h, and then washed three times. The chemiluminescence signals were detected by an ImageQuant LAS4000 mini (GE Healthcare Life Sciences, Piscataway, NJ, United States) and analyzed by ImageJ software.

### Statistical Analysis

Data were presented as the mean ± SD and analyzed using a one-way analysis of variance (ANOVA). The Tukey test was used for multiple comparisons. GraphPad Prism 5.0 software was employed to perform statistical analysis, and *p* < 0.05 was considered statistically significant.

## Results

### *Ligusticum chuanxiong* Hort Plays a Major Role in Improving Behavior Test in the Combination Therapy

**FIGURE 1:**
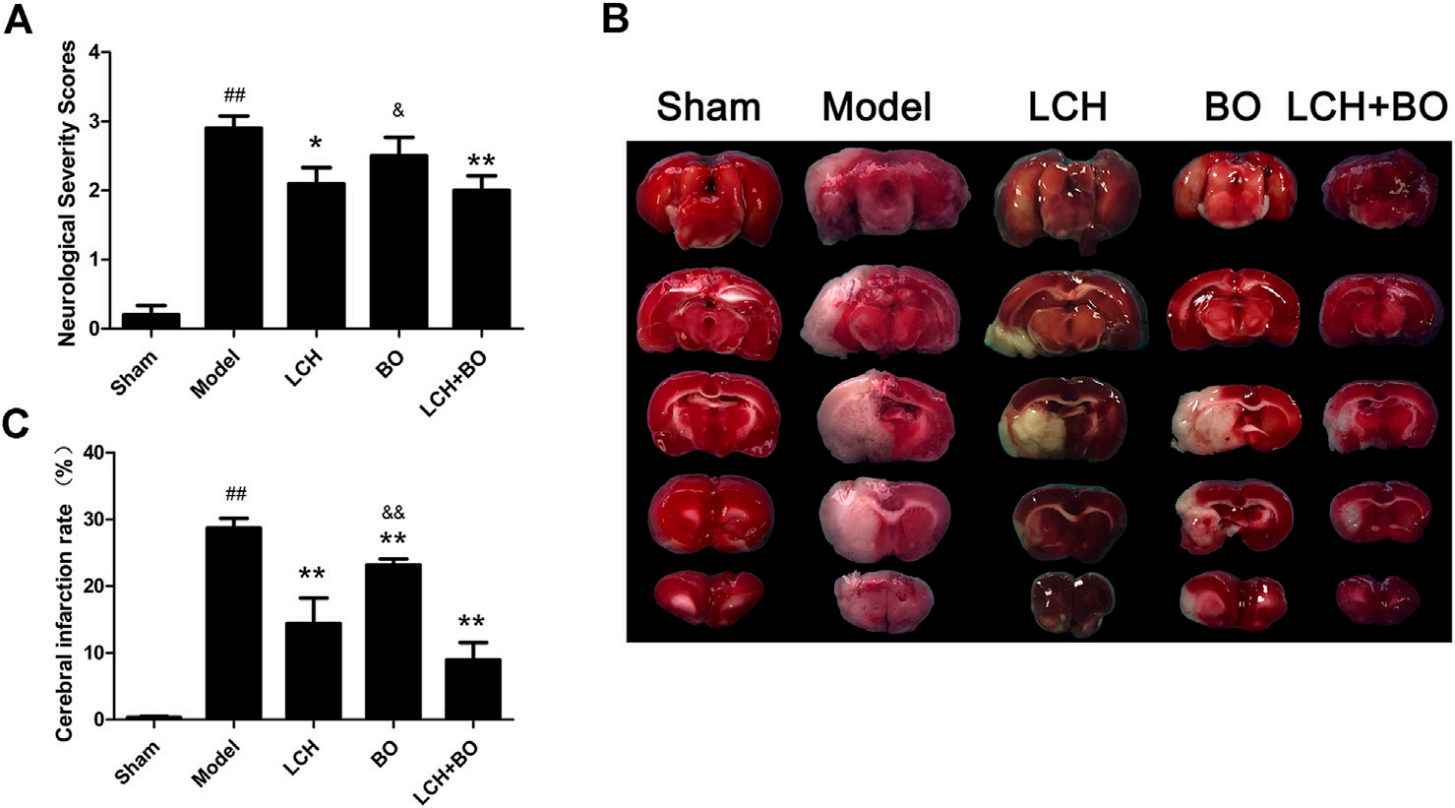
Neurological severity scores and cerebral infarction rate of MCAO rats. **(A)** The results of neurological severity scores (*n* = 10). **(B)** Representative pictures of TTC staining. **(C)** The results of cerebral infarction ratio (*n* = 6). ^##^ *p* < 0.01 compared to the sham group; ^*^ *p* < 0.05, ^**^ *p* < 0.01 compared to the model group; ^&^ *p* < 0.05, ^&&^ *p* < 0.01 compared to the LCH + BO group.

As shown in Figure 1A, MCAO model rat displayed significant increase of NSS in comparison with the sham group (*p* < 0.01), which suggested that cerebral ischemia induced abnormal behavior and movement. With the single treatment of LCH, the NSS obviously reduced (*p* < 0.05), which indicated the recovery of neurological function. Although BO itself did not improve NSS, it elevated the therapeutic effect of LCH (*p* < 0.01). Additionally, the LCH + BO group exhibited a better performance than the BO group. These above results provided evidence for the synergy treatment of LCH and BO on cerebral ischemia, and confirmed the major role of LCH in behavior improvement during the combined therapy.

### *Ligusticum Chuanxiong* Hort and Borneol Synergically Reduce Infarct Areas

TTC can dye normal brain tissue to red, while the ischemic region maintains white (Figure 1B). In the present study, LCH and BO markedly decreased infarct brain areas of MCAO rats (*p* < 0.01), which indicated that both of them ameliorated ischemia attack (Figure 1C). Interestingly, their combination exhibited a much more powerful protection than BO monotherapy (*p* < 0.01), which implied that the potential mechanisms of their protections might be different.

### *Ligusticum chuanxiong* Hort and Borneol Synergically Attenuate the Loss of Neurons in Dentate Gyrus and Subventricular Zone Regions

**FIGURE 2:**
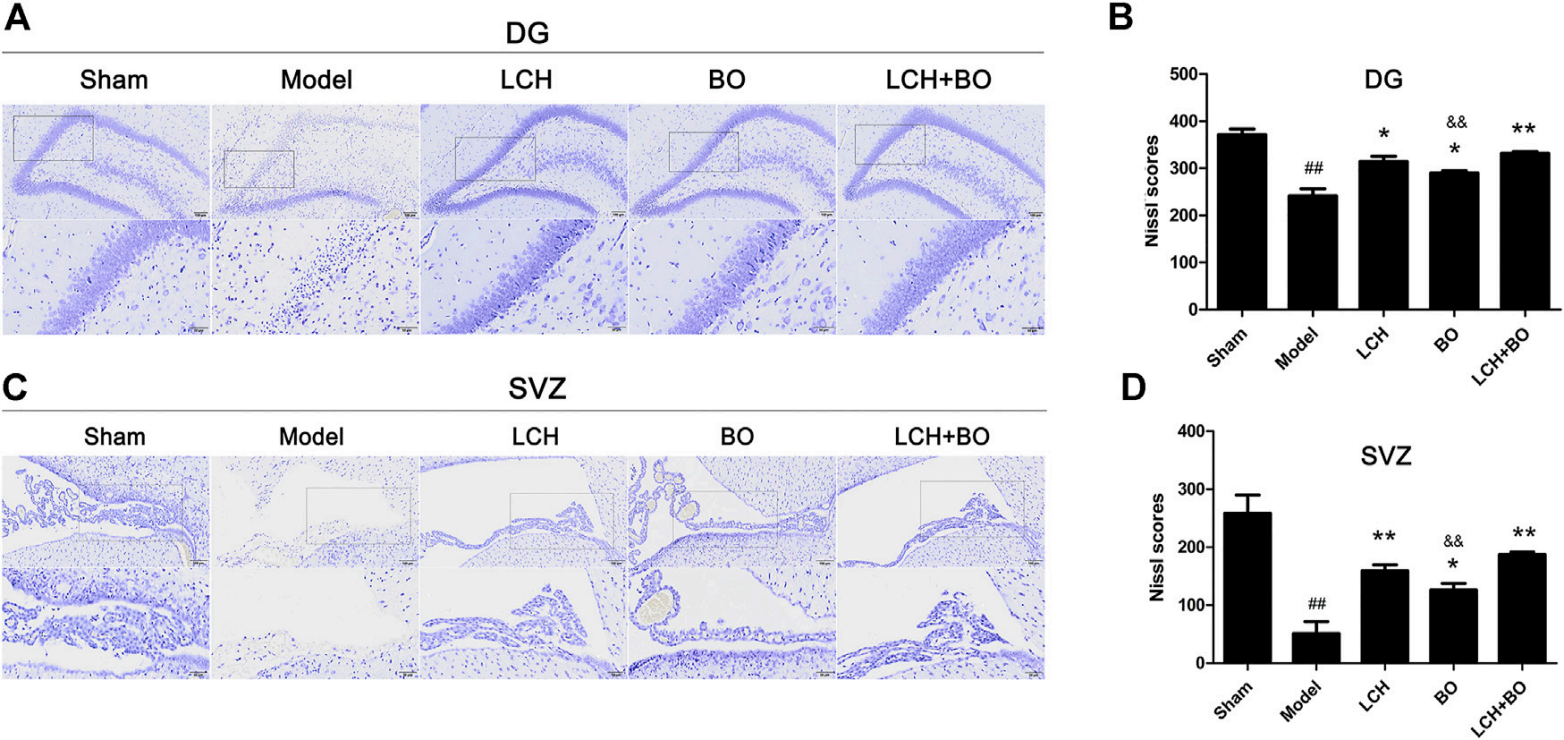
Nissl scores within DG and SVZ of MCAO rats. **(A)** Representative pictures of Nissl staining in the DG area. **(B)** The results of Nissl scores in the DG area (*n* = 3). **(C)** Representative pictures of Nissl staining in the SVZ area. **(D)** The results of Nissl scores in the SVZ area (*n* = 3). ^##^ *p* < 0.01 compared to the sham group; ^*^ *p* < 0.05, ^**^ *p* < 0.01 compared to the model group; ^&&^ *p* < 0.01 compared to the LCH + BO group.

The morphological trait of Nissl bodies reflected the statue of neurons. Obviously, MCAO attack led to extensive death of neurons in both DG (Figures 2A,B) and SVZ regions (Figures 2C,D). Although both LCH and BO exhibited their improvements on Nissl scores (*p* < 0.05, 0.01), their degrees of protection were different. LCH displayed better maintenances on shape and amount of Nissl bodies than BO in both of the regions. Moreover, the score of the LCH + BO group was much more than that of the BO group (*p* < 0.01). Apparently, LCH played a major role in neuron maintenance during the combined therapy.

### *Ligusticum chuanxiong* Hort Displays Significant Advantages on Neural Stem Cell Proliferation and Neurogenesis

**FIGURE 3:**
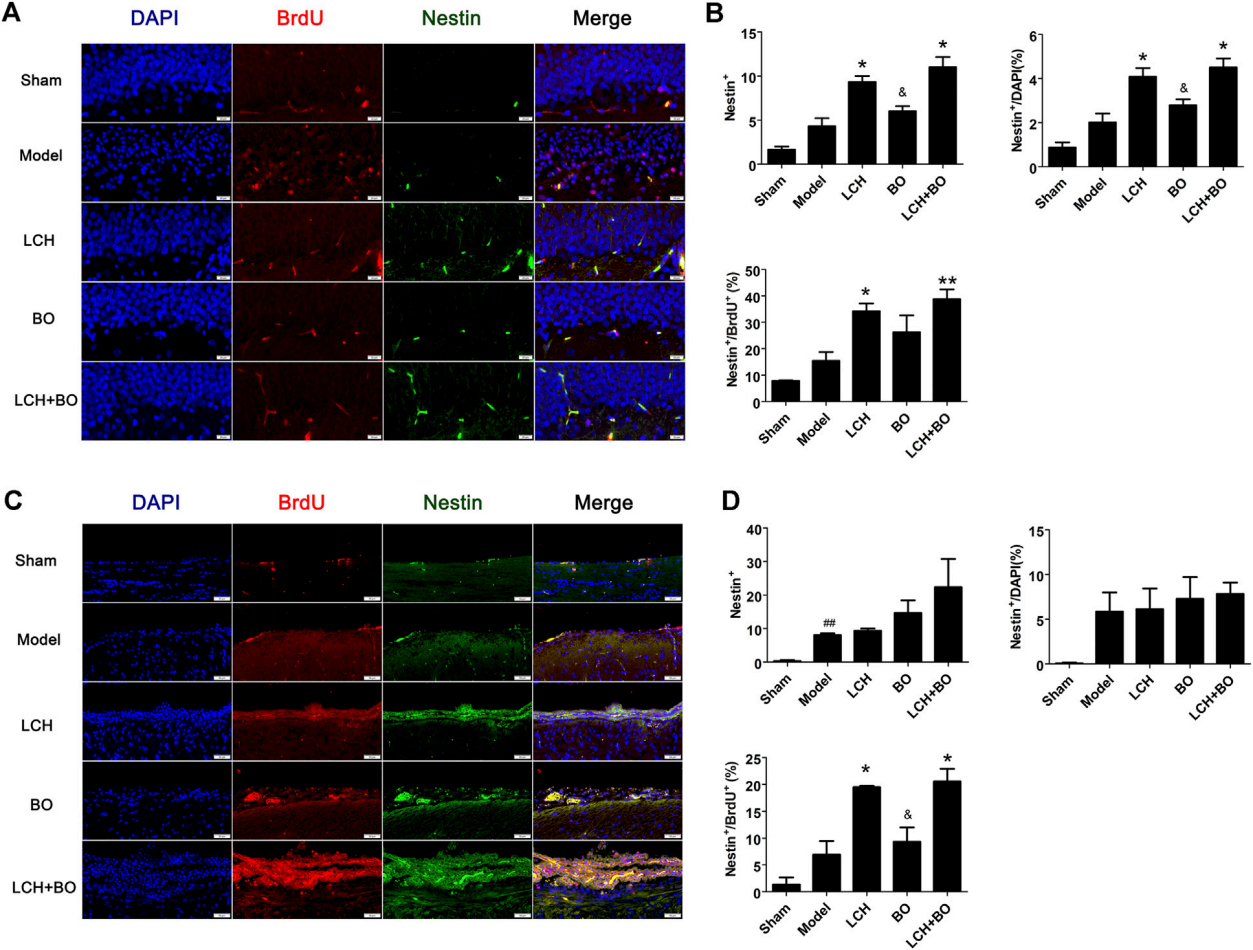
NSC proliferation within DG and SVZ of MCAO rats. **(A)** Representative double-immunostaining images of Nestin^+^/BrdU^+^ in the DG area. **(B)** The results of Nestin^+^ cells, Nestin^+^/DAPI, and Nestin^+^/BrdU^+^ in the DG area (*n* = 3). **(C)** Representative double-immunostaining images of Nestin^+^/BrdU^+^ in the SVZ area. **(D)** The results of Nestin^+^ cells, Nestin^+^/DAPI, and Nestin^+^/BrdU^+^ in the SVZ area (*n* = 3). ^##^ *p* < 0.01 compared to the sham group; ^*^ *p* < 0.05, ^**^ *p* < 0.01 compared to the model group; ^&^ *p* < 0.05 compared to the LCH + BO group.

**FIGURE 4:**
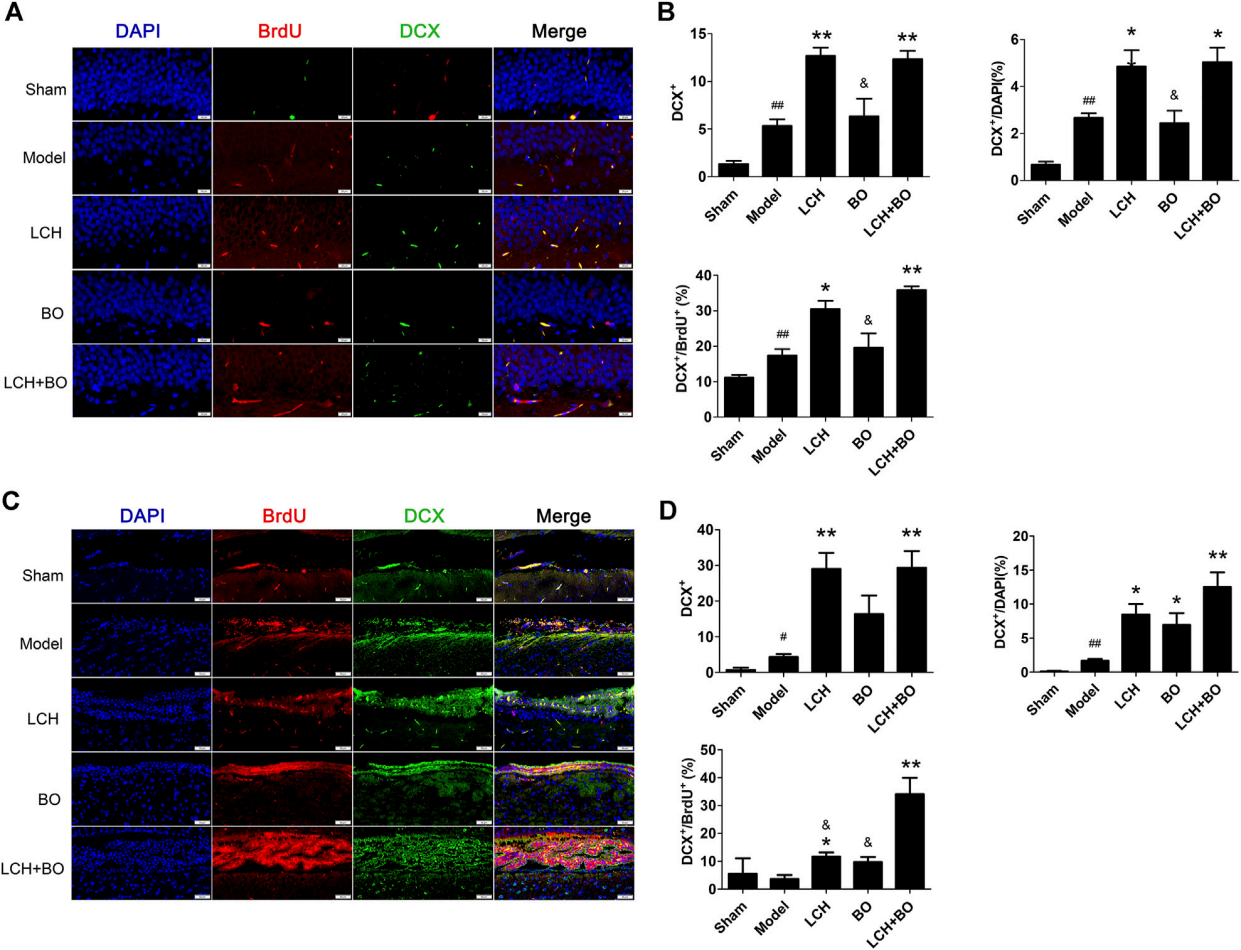
Neurogenesis within DG and SVZ of MCAO rats. **(A)** Representative double-immunostaining images of DCX^+^/BrdU^+^ in the DG area. **(B)** The results of DCX^+^ cells, DCX^+^/DAPI, and DCX^+^/BrdU^+^ in the DG area (*n* = 3). **(C)** Representative double-immunostaining images of DCX^+^/BrdU^+^ in the SVZ area. **(D)** The results of DCX^+^ cells, DCX ^+^/DAPI, and DCX ^+^/BrdU^+^ in the SVZ area (*n* = 3). ^#^ *p* < 0.05, ^##^ *p* < 0.01 compared to the sham group; ^*^ *p* < 0.05, ^**^ *p* < 0.01 compared to the model group; ^&^ *p* < 0.05 compared to the LCH + BO group.

The co-localizations of Nestin^+^/BrdU^+^ and DCX^+^/BrdU^+^ were detected to explore the synergic mechanism between LCH and BO on NSC proliferation and neurogenesis in the present study. It was demonstrated that ischemic stroke increased Nestin^+^ cells in SVZ (Figures 3C,D) and DCX^+^/DAPI in both SVZ and DG (Figure 4) (*p* < 0.01). Apparently, the hypoxic condition induced proliferations of NSCs and neurogenesis, which was similar to those of previous reports (Lin et al., 2015; Huang et al., 2020).

LCH increased Nestin^+^, Nestin^+^/DAPI, and Nestin^+^/BrdU^+^ in DG, and Nestin^+^/BrdU^+^ in SVZ (*p* < 0.05), which implied that LCH promoted the proliferation of NSCs (Figure 3). Although BO showed no effect on above indexes, it further elevated the effect of LCH on Nestin^+^/BrdU^+^ in DG (*p* < 0.01). In the assessments of neurogenesis (Figure 4), LCH increased DCX^+^ cells, DCX^+^/DAPI, and DCX^+^/BrdU^+^ in the two regions, while BO only enhanced DCX^+^/DAPI in SVZ (*p* < 0.05,0.01). Besides, their combined treatment showed more obvious improvements on DCX^+^/BrdU^+^ in both the areas. The results suggested that LCH played key roles in NSC proliferation and neurogenesis, while BO further improved the effects of LCH to some extent.

### *Ligusticum chuanxiong* Hort Plays an Important Role in Neuron Survival and Astrocyte Proliferation

**FIGURE 5:**
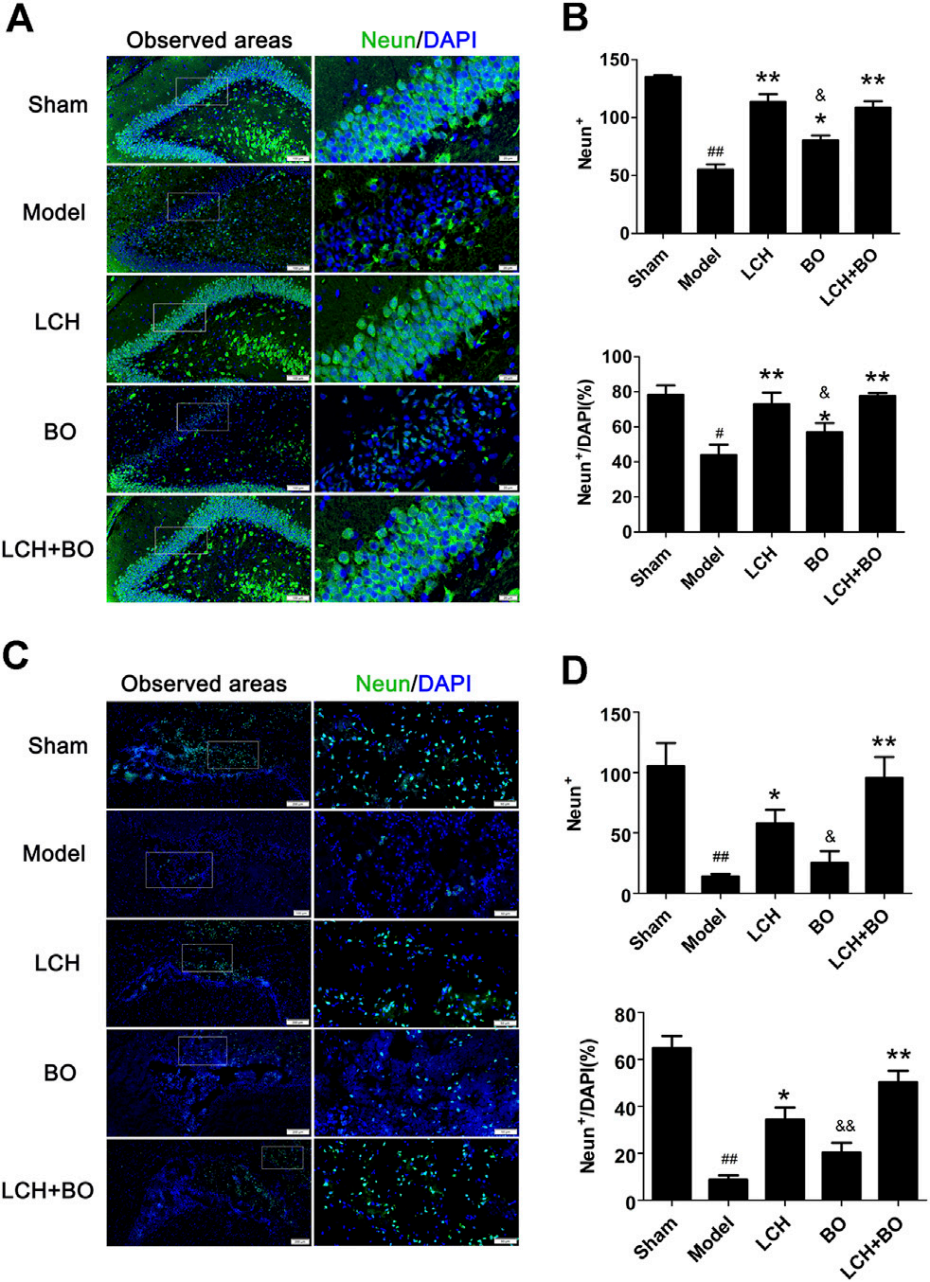
Mature neuron preservation within DG and SVZ of MCAO rats. **(A)** Representative immunostaining images of NeuN in the DG area. **(B)** The results of NeuN^+^ cells and NeuN^+^/DAPI in the DG area (*n* = 3). **(C)** Representative immunostaining images of NeuN in the SVZ area. **(D)** The results of NeuN^+^ cells and NeuN^+^/DAPI in the SVZ area (*n* = 3). ^#^ *p* < 0.05, ^##^ *p* < 0.01 compared to the sham group; ^*^ *p* < 0.05, ^**^ *p* < 0.01 compared to the model group; ^&^ *p* < 0.05, ^&&^ *p* < 0.01 compared to the LCH + BO group.

**FIGURE 6:**
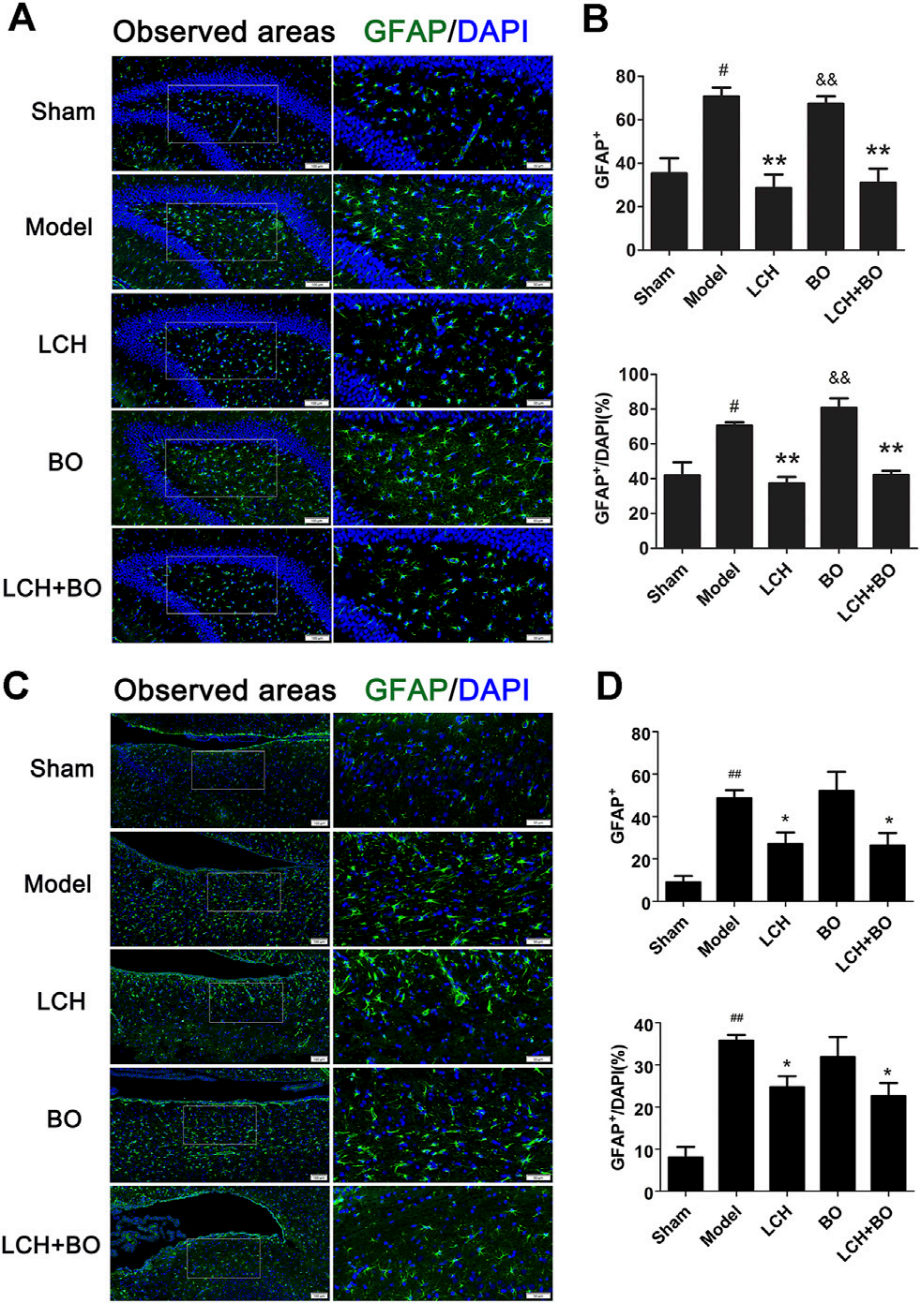
ACs proliferation within DG and SVZ of MCAO rats. **(A)** Representative immunostaining images of GFAP in the DG area. **(B)** The results of GFAP^+^ cells and GFAP^+^/DAPI in the DG area (*n* = 3). **(C)** Representative immunostaining images of GFAP in the SVZ area. **(D)** The results of GFAP^+^ cells and GFAP^+^/DAPI in the SVZ area (*n* = 3). ^#^ *p* < 0.05, ^##^ *p* < 0.01 compared to the sham group; ^*^ *p* < 0.05, ^**^ *p* < 0.01 compared to the model group; ^&^ *p* < 0.05, ^&&^ *p* < 0.01 compared to the LCH + BO group.

NeuN and GFAP are usually used as the markers of mature neurons and ACs, respectively. The present study found that ischemic stroke induced proliferations of ACs and loss of mature neurons. LCH increased NeuN^+^ cells and NeuN^+^/DAPI (Figure 5), and decreased GFAP^+^ and GFAP^+^/DAPI (Figure 6) in the two brain regions, while BO only enhanced NeuN^+^ cells and NeuN^+^/DAPI within the DG area (*p* < 0.05, 0.01). Besides, their combined treatment showed more increases on NeuN^+^ cells and NeuN^+^/DAPI in SVZ (*p* < 0.01). Similarly, the results suggested that LCH played main roles in preserving mature neurons and prohibiting AC proliferation, while BO strengthened the effect of LCH on neuron protection.

### Borneol Plays a Key Role in Improving Blood–Brain Barrier Function in the Combined Therapy

**FIGURE 7:**
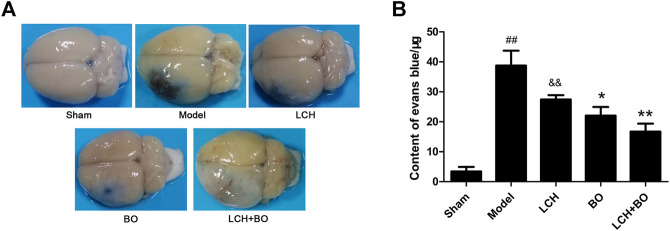
BBB permeability evaluation of MCAO rats. **(A)** Representative brain pictures after the injection of Evans blue. **(B)** The contents of Evans blue in the brains of MCAO rats (*n* = 6). ^##^ *p* < 0.01 compared to the sham group; ^*^ *p* < 0.05, ^**^ *p* < 0.01 compared to the model group; ^&&^ *p* < 0.01 compared to the LCH + BO group.

EB, as a blue dye, is regarded as an indicator of BBB function (Figure 7A). The content of EB in the model rat brain was much more than that in the sham group (Figure 7B), which indicated that MCAO injury disrupted the BBB and enhanced its permeability. Compared to the model group, the LCH group showed no obvious effect on BBB function (*p* > 0.05), while BO markedly reduced the EB content in the brain tissue (*p* < 0.05). Moreover, their synergic therapy even showed a more obvious improvement on BBB function (*p* < 0.01). Apparently, unlike the above indexes on neurological function, BO, instead of LCH, played a key role in maintaining the BBB during the combined treatment.

### Recovery of Blood–Brain Barrier Ultrastructure Owes to Borneol in the Combined Treatment

**FIGURE 8:**
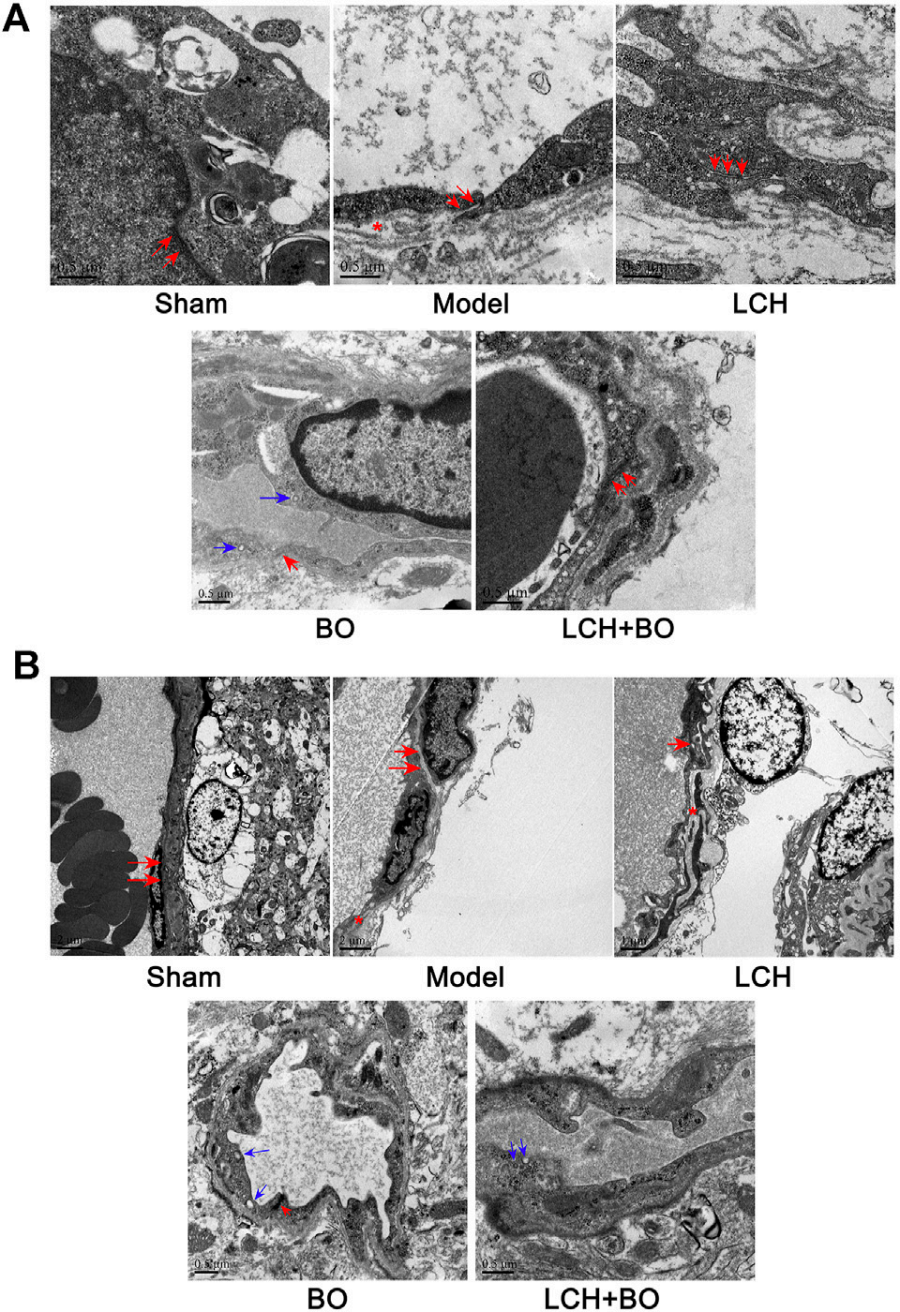
BBB ultrastructures within DG and SVZ of MCAO rats. **(A)** Representative images of transmission electron microscope within the DG area. **(B)** Representative images of transmission electron microscope within the SVZ area. The red arrows denoted TJs. The blue arrows denoted pinocytotic vesicles. The red asterisks denoted cavities between the endothelium and basement membrane.

For exploring the potential BBB maintenance mechanism of BO, a transmission electron microscope technology was adopted to observe ultrastructures of the BBB. The results in DG (Figure 8A) and SVZ (Figure 8B) were similar on the whole. For BBB structures of the sham group, the cytomembrane boundaries of BMECs were clear and smooth without obvious pinocytosis. Particularly, the TJs between endothelial cells were normal without any gap. The connections between endothelium and basement membrane were also tight.

Yet, brain ischemia attacked BBB structures extensively and deeply. In the model group, cavities emerged between the endothelium and basement membrane, and the TJs were slightly loose, which indicated that BBB structures had been damaged to some extent. No obvious pinocytosis was found yet. The BBB ultrastructures of the LCH group were similar to those of the model group.

Under the treatment of BO, the TJs between endothelial cells were restored, and the gaps between endothelium and basement membrane were significantly reduced, which indicated that BBB structures were recovering from the ischemic injury. Interestingly, some pinocytotic vesicles appeared in the cytoplasm near the vascular lumen. The results implied that BO not only improved BBB structures but also promoted pinocytosis, which might help the drugs with small molecule across the BBB and influx into the brain. The LCH + BO group had performance similar to the BO group.

### Borneol Provides a Major Effect on Brain Microvascular Endothelial cell Proliferation and Tight Junction–Associated Proteins Expressions

**FIGURE 9:**
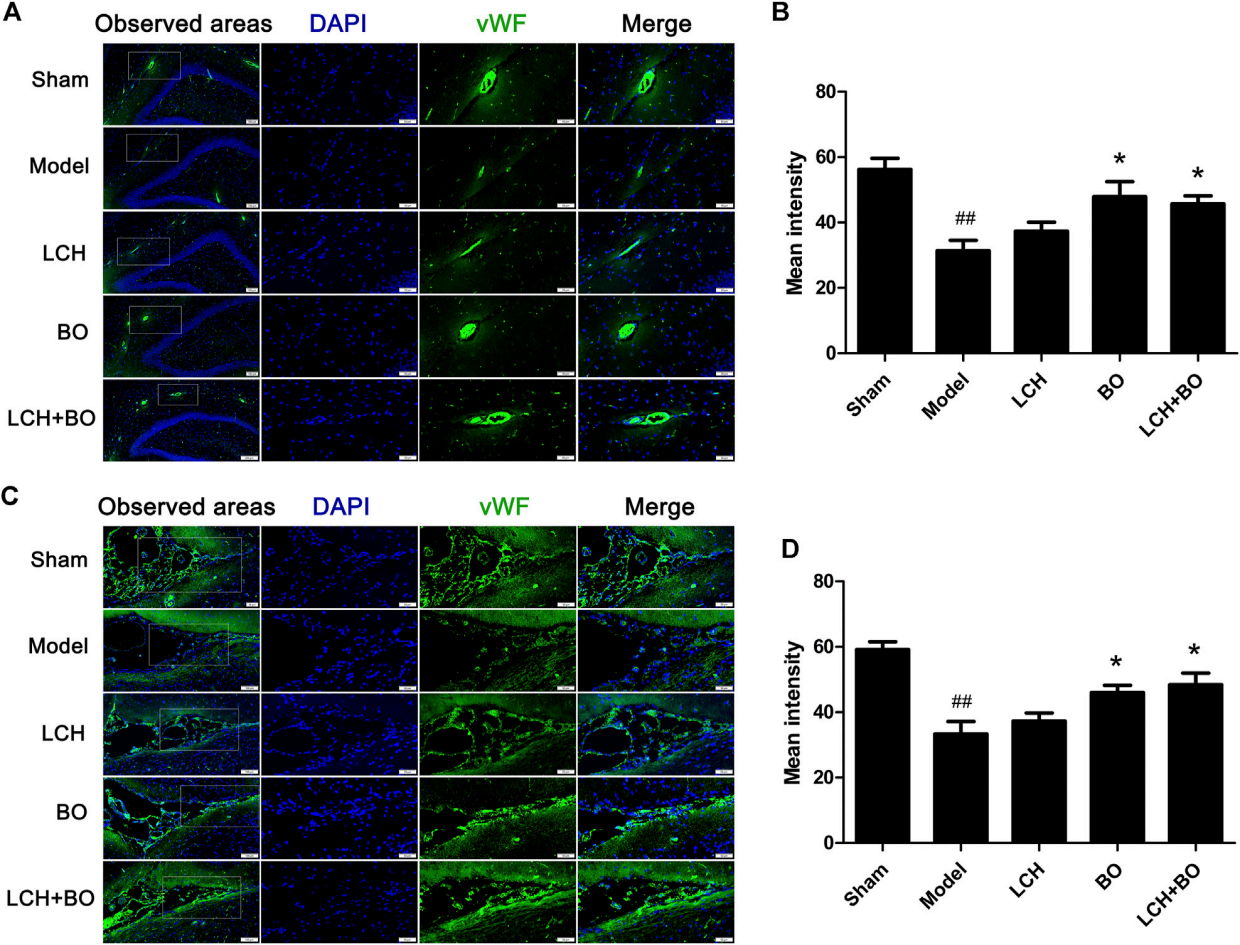
Expressions of vWF within DG and SVZ of MCAO rats. **(A)** Representative immunostaining images of vWF in the DG area. **(B)** The results of average fluorescence intensities of vWF in the DG area (*n* = 3). **(C)** Representative immunostaining images of vWF in the SVZ area. **(D)** The results of average fluorescence intensities of vWF in the SVZ area (*n* = 3). ^##^ *p* < 0.01 compared to the sham group; ^*^ *p* < 0.05 compared to the model group.

**FIGURE 10:**
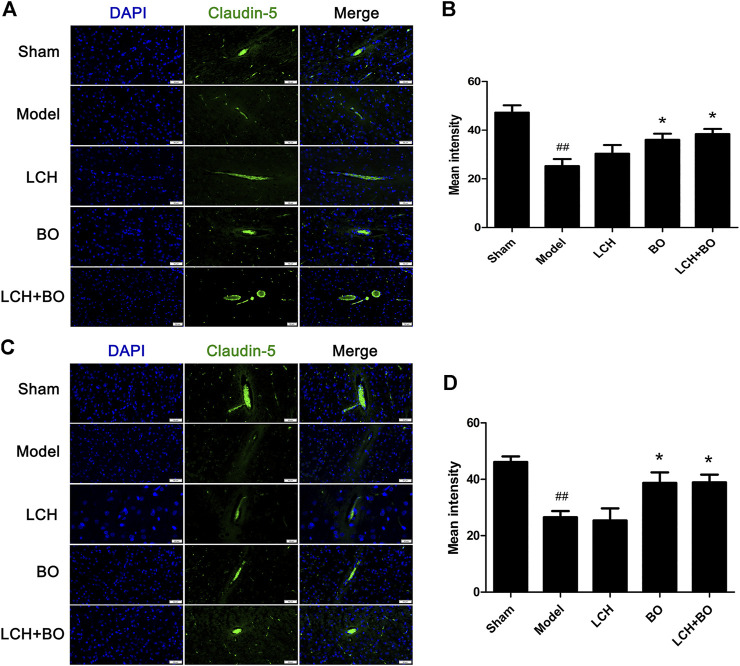
Expressions of claudin-5 within DG and SVZ of MCAO rats. **(A)** Representative immunostaining images of claudin-5 in the DG area. **(B)** The results of average fluorescence intensities of claudin-5 in the DG area (*n* = 3). **(C)** Representative immunostaining images of claudin-5 in the SVZ area. **(D)** The results of average fluorescence intensities of claudin-5 in the SVZ area (*n* = 3). ^##^ *p* < 0.01 compared to the sham group; ^*^ *p* < 0.05 compared to the model group.

**FIGURE 11:**
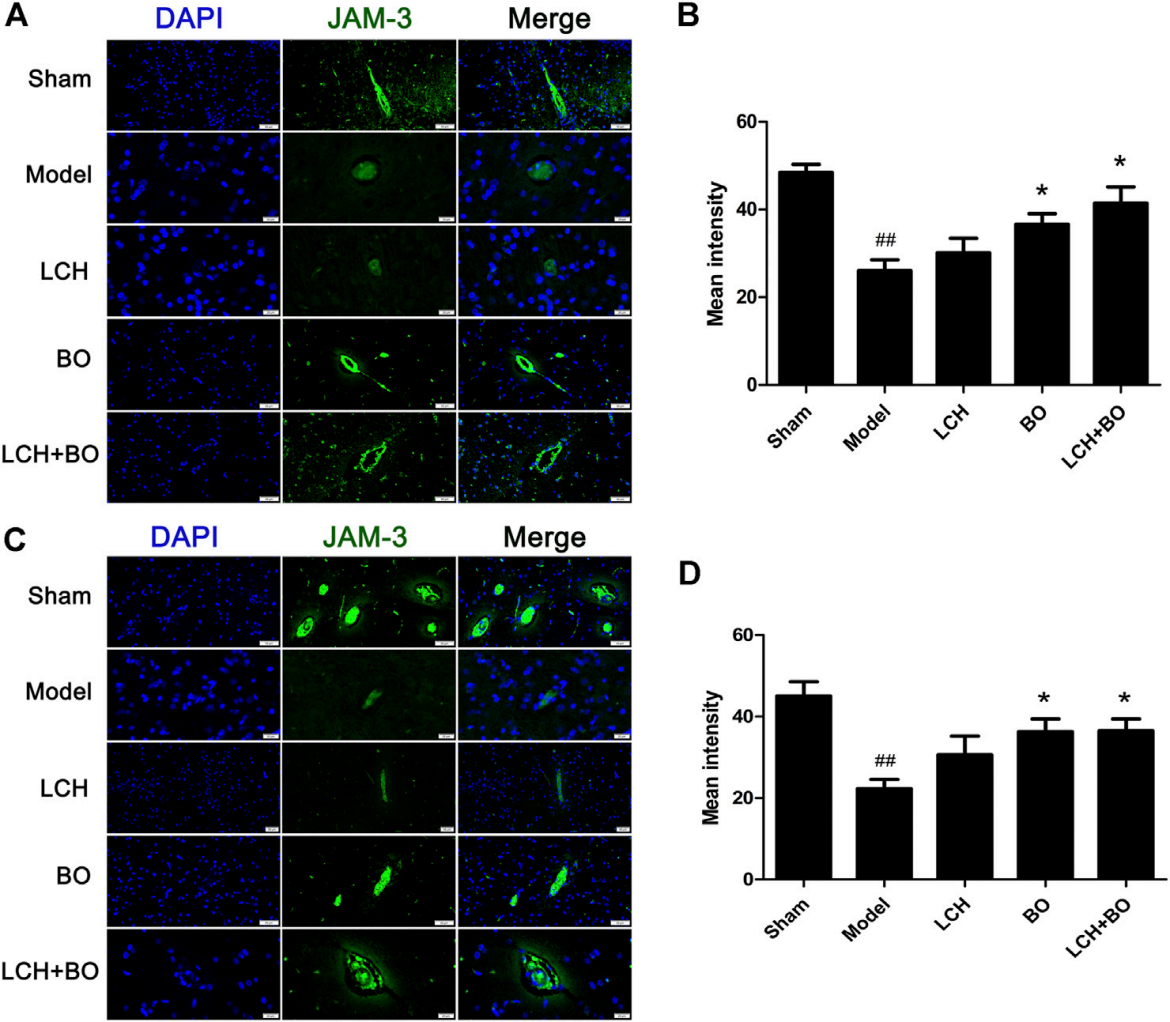
Expressions of JAM-3 within DG and SVZ of MCAO rats. **(A)** Representative immunostaining images of JAM-3 in the DG area. **(B)** The results of average fluorescence intensities of JAM-3 in the DG area (*n* = 3). **(C)** Representative immunostaining images of JAM-3 in the SVZ area. **(D)** The results of average fluorescence intensities of JAM-3 in the SVZ area (*n* = 3). ^##^ *p* < 0.01 compared to the sham group; ^*^ *p* < 0.05 compared to the model group.

**FIGURE 12:**
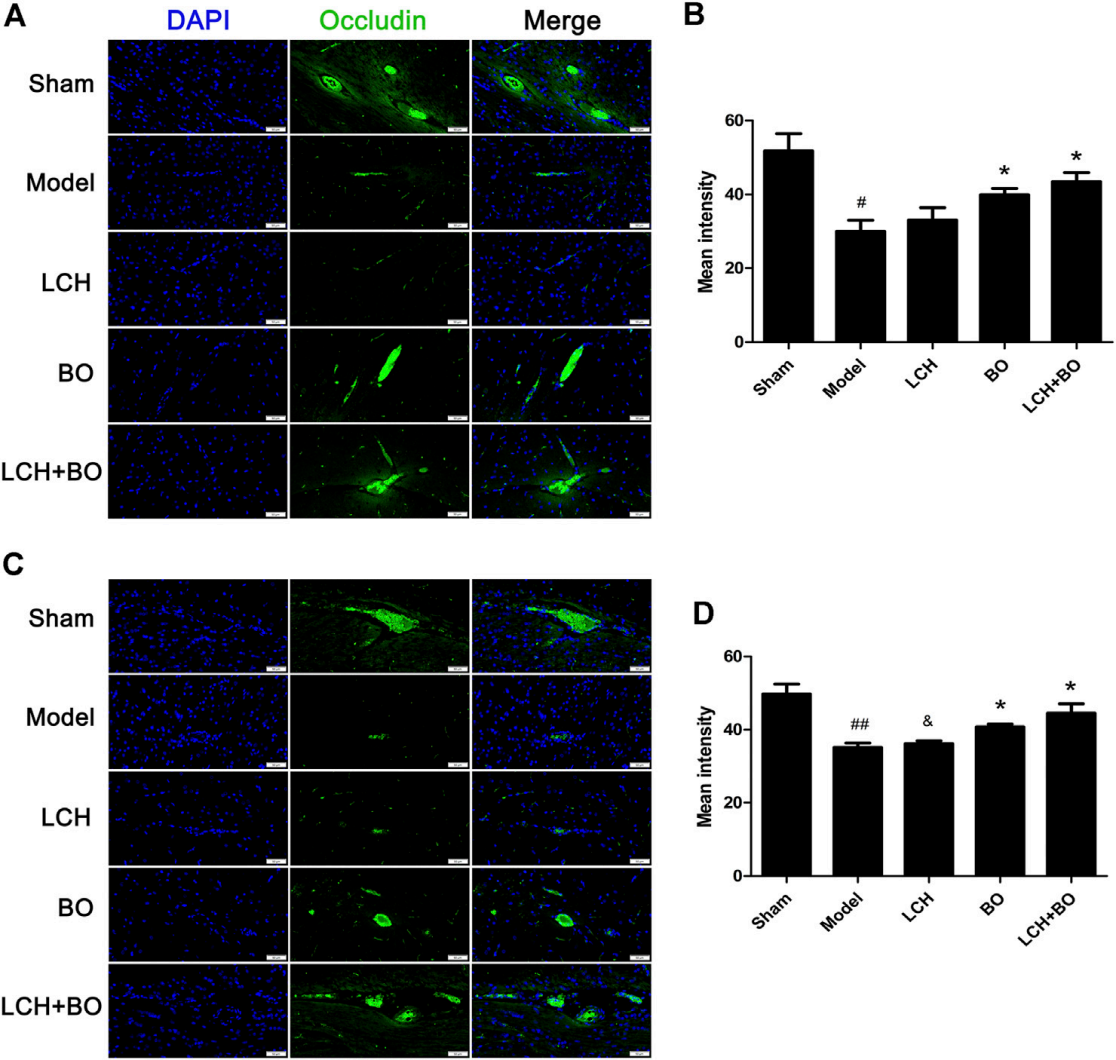
Expressions of occludin within DG and SVZ of MCAO rats. **(A)** Representative immunostaining images of occludin in the DG area. **(B)** The results of average fluorescence intensities of occludin in the DG area (*n* = 3). **(C)** Representative immunostaining images of occludin in the SVZ area. **(D)** The results of average fluorescence intensities of occludin in the SVZ area (*n* = 3). ^#^ *p* < 0.05, ^##^ *p* < 0.01 compared to the sham group; ^*^ *p* < 0.05 compared to the model group; ^&^ *p* < 0.05 compared to the LCH + BO group.

**FIGURE 13:**
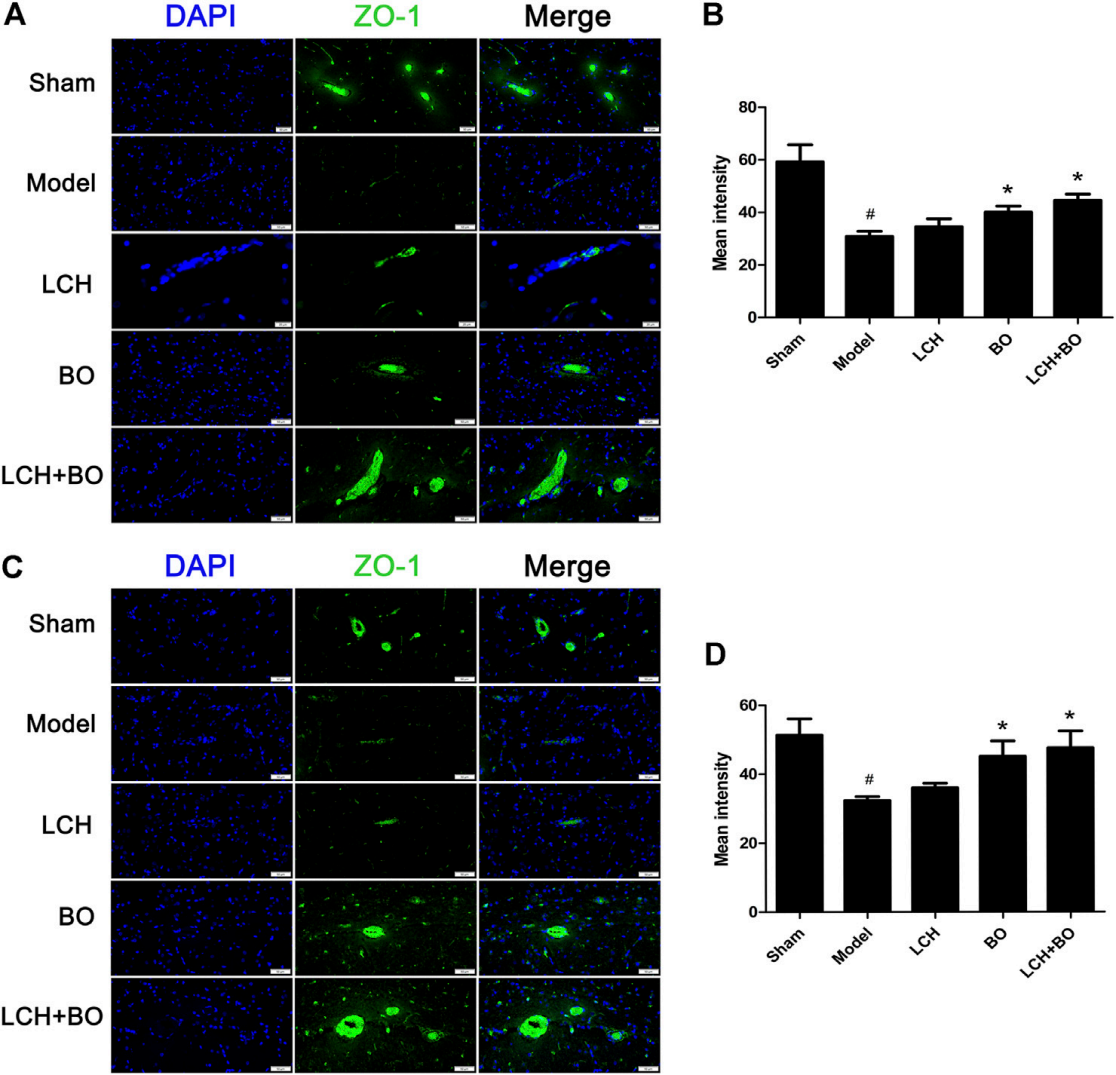
Expressions of ZO-1 within DG and SVZ of MCAO rats. **(A)** Representative immunostaining images of ZO-1 in the DG area. **(B)** The results of average fluorescence intensities of ZO-1 in the DG area (*n* = 3). **(C)** Representative immunostaining images of ZO-1 in the SVZ area. **(D)** The results of average fluorescence intensities of ZO-1 in the SVZ area (*n* = 3). ^#^ *p* < 0.05 compared to the sham group; ^*^ *p* < 0.05 compared to the model group.

von Willebrand factor is widely regarded as a biomarker of BMECs, which are sealed by TJ-associated proteins, such as claudin-5, JAM-3, occludin, and ZO-1 (Zhao et al., 2020). Compared to the sham group, the expressions of vWF (Figure 9), claudin–5 (Figure 10), JAM–3 (Figure 11), occludin (Figure 12), and ZO–1(Figure 13) in the model group were all reduced markedly (*p* < 0.05,0.01) in DG and SVZ areas, which indicated the loss of BMECs and deficiencies of TJs. Unlike the effects on neurogenesis, LCH shows no obvious improvement in BMECs and TJs proteins (*p* > 0.05). However, BO displayed surprising inhibitions on the decreases of vWF, claudin-5, JAM-3, occludin, and ZO-1 in both the areas (*p* < 0.05), which verified its advantages in maintaining the BBB structure, and further cleared its mechanisms in reducing BBB permeability and improving ultrastructure above.

### *Ligusticum chuanxiong* Hort and Borneol Display Different Focuses on the Regulations of Neurotrophins

**FIGURE 14:**
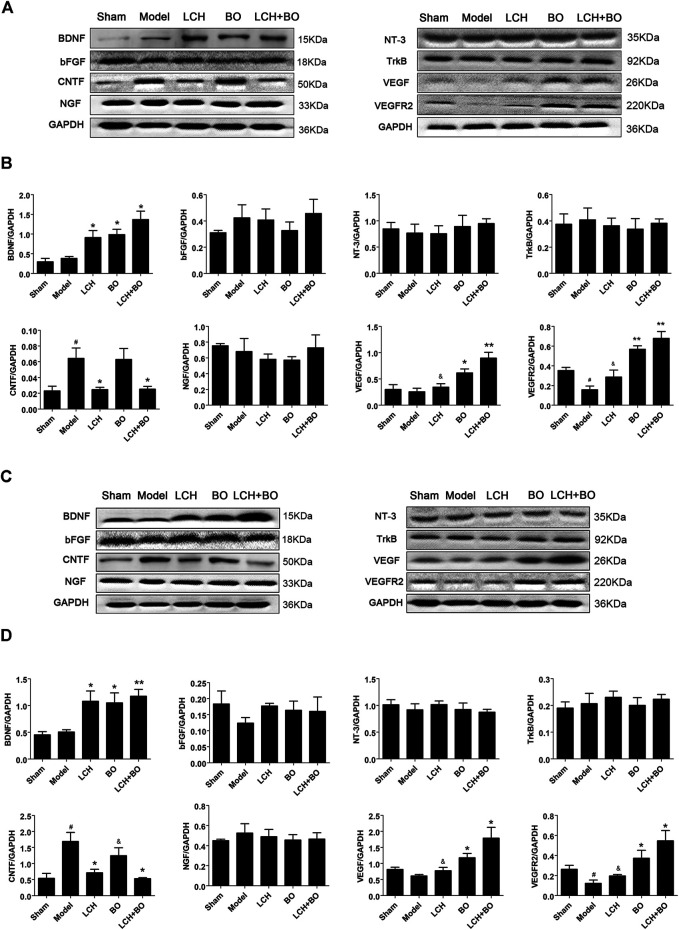
Expressions of BDNF, bFGF, CNTF, NGF, NT-3, TrkB, VEGF, and VEGFR2 within DG and SVZ of MCAO rats. **(A)** Blot images of the proteins in the DG area. **(B)** Relative expression of the proteins to GAPDH in the DG area (*n* = 3). **(C)** Blot images of the proteins in the SVZ area. **(D)** Relative expressions of the proteins to GAPDH in the SVZ area (*n* = 3). ^#^ *p* < 0.05 compared to the sham group; ^*^ *p* < 0.05, ^**^ *p* < 0.01 compared to the model group; ^&^ *p* < 0.05 compared to the LCH + BO group.

Neurotrophins play key roles in neurogenesis, and some of which are even involved in angiogenesis and BBB maintenance, such as VEGF and bFGF. In the present research, the results in DG (Figures 14A,B) were basically similar to those in SVZ (Figures 14C,D), except differences in degree. The increased CNTF and decreased VEGFR2 were shown in DG and SVZ areas of the model group (*p* < 0.05). LCH increased the expression of BDNF and decreased that of CNTF, while BO increased BDNF, VEGF, and VEGFR2 (*p* < 0.05, 0.01). Basically, the combined treatment had a superposition effect of their monotherapies, including increases of BDNF, VEGF, and VEGFR2, and decreases of CNTF. But the improvement of VEGF in the LCH + BO group (*p* < 0.01) was better than that in the BO group (*p* < 0.05), with the condition that LCH showed no effect on the protein (*p* > 0.05), which displayed their synergy to some degree.

### *Ligusticum chuanxiong* Hort is the Main Contributor in Reversing Astrocytes From A1 Phenotype to A2

**FIGURE 15:**
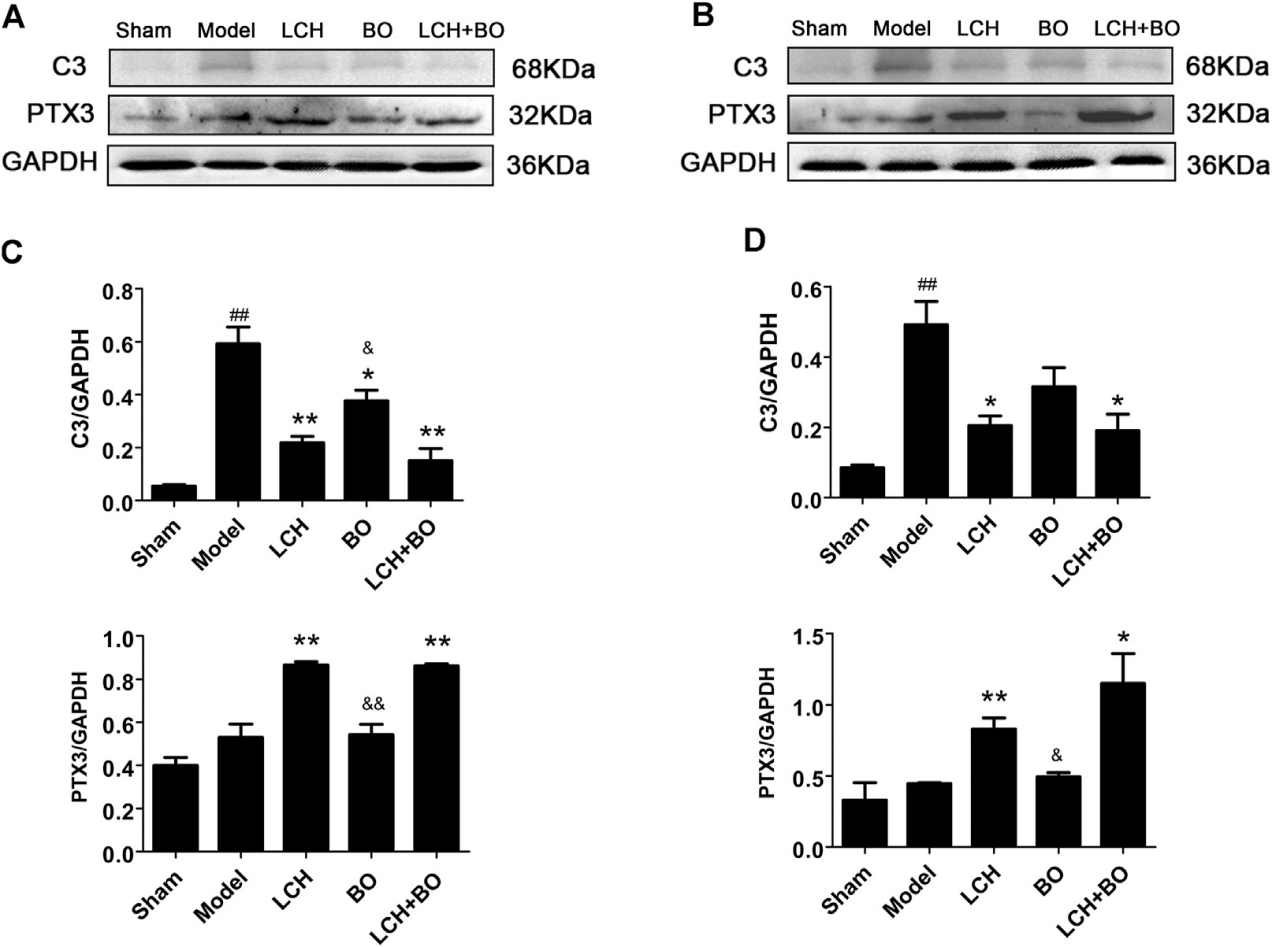
Expressions of C3 and PTX3 within DG and SVZ of MCAO rats. **(A)** Blot images of the proteins in the DG area. **(B)** Blot images of the proteins in the SVZ area. **(C)** Relative expressions of the proteins to GAPDH in the DG area (*n* = 3). **(D)** Relative expressions of the proteins to GAPDH in the SVZ area (*n* = 3). ^##^ *p* < 0.01 compared to the sham group; ^*^ *p* < 0.05, ^**^ *p* < 0.01 compared to the model group; ^&^ *p* < 0.05, ^&&^ *p* < 0.01 compared to the LCH + BO group.

C3 and PTX3 are widely used as the markers of A1 and A2 ACs, respectively (Su et al., 2019). The present research showed that ischemic injury induced the increases of C3 in both DG (Figure 15A) and SVZ (Figure 15B) areas (*p* < 0.01). LCH not only decreased C3 but also enhanced PTX3 in the two regions (Figures 15C,D) (*p* < 0.01). Surprisingly, BO also decreased C3 level in DG (*p* < 0.05). The therapeutic output of the combined treatment was similar to that of the LCH group. The results indicated that LCH provided the main regulation in reversing reactive ACs from A1 phenotype to A2 during the combined therapy.

## Discussion

According to the theory of traditional Chinese medicine, the common therapeutic principle of ischemic stroke is removing blood stasis and inducing resuscitation (Huayu Kaiqiao). LCH and BO are the representative medicines of above therapies, respectively, and widely used for cerebral ischemia patients as a combination, such as Huatuo Zaizao formula and Naoxintong formula (Su et al., 2011) (Duan et al., 2017). Our previous studies also confirmed their synergy in alleviating the loss of neurons and damage of BMECs in cerebral ischemic rats (Yu et al., 2020a; Yu et al., 2020b). However, it is still unclear whether the excellent output of their combined treatment is related to their synergic regulations on neurogenesis and BBB maintenance.

Uncoordinated movement of the limbs or trunk is a typical symptom of stroke, and this abnormal behavior is positively correlated with the degree of neuron injury (Tanaka et al., 2018). In the present study, single LCH treatment markedly enhanced NSS, which exhibited the advantage of LCH in restoring neurological output. Although both LCH and BO significantly reduced infarct ratios and elevated Nissl scores, their combination displayed a much better effect than their monotherapies on the above indexes. Generally, it is difficult for the combination of drugs with a similar mechanism to bring much better therapeutic effect than their monotherapies. Thus, we inferred that there might be different mechanisms between LCH and BO on their attenuation of ischemic injury. Then the following results verified our hypothesis.

In the results of immunofluorescence measurements, LCH obviously increased the ratio of Nestin^+^/BrdU^+^, which indicated that it upregulated the proliferative potential of NSCs. Moreover, LCH increased DCX^+^/BrdU^+^, DCX^+^/DAPI, and NeuN^+^/DAPI, and decreased GFAP^+^/DAPI, which indicated that LCH might be involved in inhibiting the excessive reactive astrogliosis, and modulating neurogenesis by guiding the differentiation of NSCs toward neurons, instead of ACs. Additionally, LCH also exerted its neuroprotective effect by alleviating the loss of mature neurons, which is similar to previous reports (Cheng et al., 2008; Gim et al., 2013; Ip et al., 2016; Gu et al., 2020). These above evidences suggested that LCH had the potential of promoting neurogenesis, which might be its new mechanism in ameliorating ischemic brain injury.

BO itself showed little effect on NSC proliferation and neurogenesis in this study. Nevertheless, the regulations on DCX^+^/DAPI, NeuN^+^, NeuN^+^/DAPI, and GFAP^+^ were further augmented in the LCH + BO group than those in the LCH group. Although BO has been confirmed to be a p-glycoprotein inhibitor and increases distributions of many drugs in the brain (Yu et al., 2013a; Yu et al., 2013b), its brain-targeting effect is meaningless when the BBB structure is destructed during cerebral ischemia. In view of this, there might be other underlying mechanisms on its neurogenesis assistance for LCH. There have been numerous reports verifying that BO is able to reduce the permeability of the BBB suffering from ischemic injury (Ni et al., 2011; Zhang et al., 2017b). However, the exact mechanism is unknown till now. The present study found that BO decreased the delivery of EB in ischemic brain, as reported previously (Chen et al., 2019). Consequently, the ultrastructure results showed that BO not only reinforced structures of the BBB TJs but also gave rise to pinocytosis, which might be another mechanism of BO in helping drugs across the BBB. The BBB is composed of three cellular elements, including endothelial cells, astrocyte end feet, and pericytes. But only endothelial cells are considered to be the most important element in the BBB structure because TJs are regarded as a series of fusion points between the membrane of adjacent cells and regulated by TJ-associated proteins, such as claudin-5, JAM-3, occludin, and ZO-1 (Ballabh et al., 2004). The protein of vWF is usually used as a biomarker of BMECs. In the model group, the downregulation of vWF indicated the loss of BMECs, which reversely led to the damage of the BBB structure. The expression of vWF was increased, and the ultrastructure of TJs was restored in both DG and SVZ areas after BO treatment. The results indicated that BO had the ability to induce the proliferation of BMECs and even repair the damaged structure of TJs, which might be a potential mechanism of BO in improving the BBB of ischemic brain. For further exploring the statuses of TJs, the immunofluorescence intensities of TJ-associated proteins were detected. Basically, the expressions of those proteins were similar between DG and SVZ areas. Ischemic attack induced the downregulations of claudin-5, JAM-3, occludin, and ZO-1, which implied the decreases of fusion points between the membrane of adjacent BMECs. Then the increased distance between adjacent BMECs caused the collapse of the BBB structure. While the abundances of claudin-5, JAM-3, occludin, and ZO-1 were all increased after BO treatment, they showed no obvious elevation in the LCH group. Apparently, BO played a more crucial role than LCH in the maintenances of the BBB structure and TJ function during the combined therapy.

It has been verified that neurotrophins play a vital role in NSC proliferation, neurogenesis, neuroplasticity, and even BBB remodeling (Maejima et al., 2019; Yang et al., 2020). The present study showed that both LCH and BO upregulated the expression of BDNF. Furthermore, LCH induced the decrease of CNTF, while BO led to the increases of VEGF and VEGFR2. However, the other neurotrophins showed no obvious alterations. BDNF, as a member of neurotrophin family, produces its physiological effect *via* binding to its receptor TrkB, dimerization of ligand and receptor, and autophosphorylation (Yang et al., 2020). There was no marked alteration on the expression of TrkB after the treatment of LCH or BO, which indicated that phosphorylation might be involved in its modulation, instead of abundance. VEGFR2 (KDR/Flk-1), with the structures of seven extracellular immunoglobulin homology domains, a transmembrane domain, and a tyrosine kinase domain, is considered to be the most important receptor of VEGF in regulating physiological and pathological angiogeneses (Takahashi et al., 1999; Simons et al., 2016; Nascimento et al., 2021). Unlike TrkB, the present research proved that the abundances of VEGFR2 and its ligand of VEGF could both be enhanced by BO in DG and SVZ areas, which might be its mechanism involved in BBB remodeling *via* angiogenesis.

Normally, ACs act as important components of neurogenic microenvironment and play an important part in neuronal maturation and function. However, stroke attack causes the loss of neurons, excessive proliferation of ACs, and even formation of glial scar within ischemic area. Reactive astrogliosis is regarded as the main source of glial scar. CNTF is able to lead to sustained AC activation, which produces multifaceted functions in CNS pathogenesis process, including beneficial and harmful aspects. However, the benefits of reactive ACs at the acute phase of stroke might be offset by its potential of negatively regulating neurogenesis at a later phase (Wilhelmsson et al., 2012). A previous study confirmed that MCAO injury led to transient increase of ACs with A2 phenotype, followed by rapid reduction, while inflammation caused increase of A1 ACs (Zamanian et al., 2012). Additionally, inflammatory reaction accompanied and aggravates the development of ischemic pathology, especially during the later stage of stroke (Kawabori and Yenari, 2015). In the present study, model rat showed significant increases of GFAP and C3, which indicated the activation of excessive A1 ACs by CNTF at the later stage of stroke, while LCH not only alleviated the activation to a certain extent *via* reducing the expression of CNTF but also guided the transformation of reactive ACs from A1 to A2, which reversely promoted the release of some neurotrophic factors, such as BDNF. Furthermore, BO also reduced reactive astroliosis with A1 phenotype *via* maintaining homeostatic intracerebral environment. Apparently, both LCH and BO had the ability of regulating ACs phenotypes, but the involved mechanisms were different.

Presently, the treatment of ischemic stroke is still limited in clinic. Inducing neurogenesis and restoring the damaged neurological function, as potential strategies, share more and more concerns. The results in this study indicated that the synergy between LCH and BO is mainly based on neurogenesis *via* transformation of AC phenotypes, modulation of neurotrophins, proliferation of NSCs, and maintenance of the BBB. Although the present study verified the underlying mechanism of LCH on neurogenesis, the main active ingredients and the synergic mechanism among these ingredients are still unclear, and seeking answers to these questions is our future work.

## Conclusion

In the present study, the synergic therapies between LCH and BO were shown on MCAO rats, including NSS, infarct areas, and Nissl scores. However, these two medicines displayed different focuses. Specifically, LCH addressed the regulations on NSC proliferation, neurogenesis, mature neurons protection, and AC transformation from A1 phenotype to A2, which then regulated the expressions of CNTF and BDNF. But BO was mainly responsible for maintaining the integrity of the BBB, including remolding structures of TJs and upregulating TJ-associated proteins. Additionally, the results also indicated that a homeostatic intracerebral environment played a crucial role in post-stroke neuroregeneration.

## Data Availability

The raw data supporting the conclusion of this article will be made available by the authors, without undue reservation.

## Abbreviations

ACs: astrocytes, BBB: blood–brain barrier, bFGF: basic fibroblast growth factor, BMECs: brain microvascular endothelial cells, BO: borneol, BSA: bovine serum albumin, C3: complement component 3, CNS: central nervous system, CNTF: ciliary neurotrophic factor, DAPI: 4′,6–diamidino–2–phenylindole, DCX: doublecortin, DG: dentate gyrus, GAPDH: glyceraldehyde-3–phosphate dehydrogenase, GC–MS: gas chromatography–mass spectroscopy, GFAP: glial fibrillary acidic protein, JAM–3: junctional adhesion molecule 3, LCH: *Ligusticum chuanxiong* Hort, MCAO: middle cerebral artery occlusion, NeuN: neuronal nuclei, NGF: nerve growth factor, NSCs: neural stem cells, NSS: neurological severity scores, PTX3: pentraxin 3, PVDF: polyvinylidene fluoride, RIPA: radio-immunoprecipitation assay, rtPA: recombinant tissue plasminogen activator, SDS–PAGE: sodium dodecyl sulfate–polyacrylamide gel electrophoresis, SVZ: subventricular zone, TBST: tris-buffered saline Tween, TJs: tight junctions, TN–3: neurotrophin-3, TrkB: tyrosine kinase receptor B, TTC: 2,3,5–triphenyltetrazolium chloride, UPLC–MS/MS: ultra-performance liquid chromatography–tandem mass spectrometry, VEGF: vascular endothelial growth factor, VEGFR2: vascular endothelial growth factor receptor 2, vWF: von Willebrand factor, ZO–1: Zonula occludens–1.
