# Supplementary material for: Synergic Neuroprotection Between Ligusticum Chuanxiong Hort and Borneol Against Ischemic Stroke by Neurogenesis via Modulating Reactive Astrogliosis and Maintaining the Blood–Brain Barrier
Source: Front Pharmacol. 2021 Jun 16;12:666790. doi: 10.3389/fphar.2021.666790 (PMC8242197; doi:10.3389/fphar.2021.666790)
Supplement: Supplementary file 1 [file DataSheet1.docx]

Supplementary Material

# 1 Result of UPLC–MS/MS Based Qualitative Analysis of LCH injection

A total of 59 components were identified in LCH injection using UPLC–MS/MS analysis. The retention time of Ligustilide, as the component of quality control of *Ligusticum chuanxiong* Hort., were 16.05672 min and 14.9955 min in positive and negative ion modes, respectively.


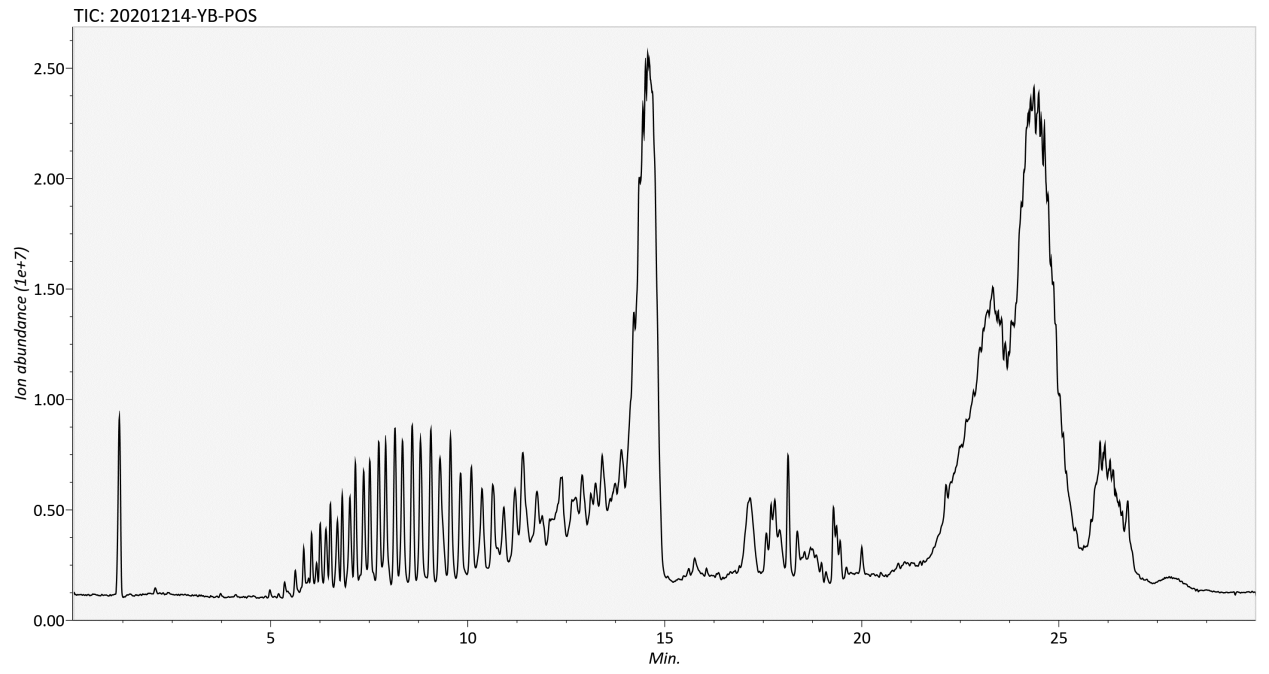


Figure S1. UPLC−MS chromatogram of LCH injection in positive ion mode


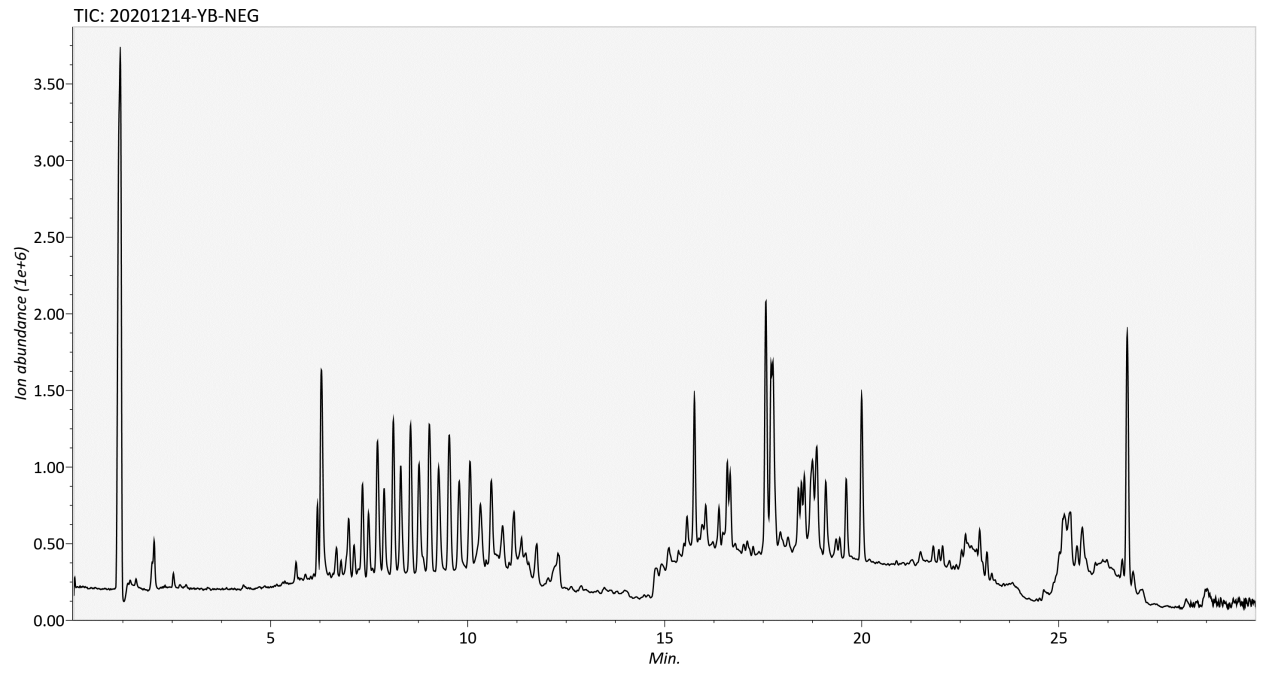


Figure S2. UPLC−MS chromatogram of LCH injection in negative ion mode

Table S1. Identified components from LCH injection by UPLC−MS/MS

| **No.** | **RT (min)** | **Adduct** | **Name** | **m/z** | **Formula** |
| --- | --- | --- | --- | --- | --- |
| 1 | 1.021083 | [M+H]^+^ | delta-dodecalactone | 199.1675 | C_12_H_22_O_2_ |
| 2 | 1.1669 | [M+H]^+^ | Ferulic Acid | 216.9226 | C_10_H_10_O_4_ |
| 3 | 2.186683 | [M+H]^+^ | 4-Hydroxy-3,6-dimethylpyran-2-one | 141.0545 | C_7_H_8_O_3_ |
| 4 | 4.1156 | [M+H]^+^ | 4`-Aminoacetanilide | 151.0963 | C_8_H_10_N_2_O |
| 5 | 5.448884 | [M+H]^+^ | Curcumin | 407.1908 | C_21_H_20_O_6_ |
| 6 | 5.5507 | [M+H]^+^ | Vanillin | 153.0537 | C_8_H_8_O_3_ |
| 7 | 5.658867 | [M+H]^+^ | Umbelliferone | 163.038 | C_9_H_6_O_3_ |
| 8 | 5.89085 | [M+H]^+^ | Cuminyl alcohol | 133.1005 | C_10_H_14_O |
| 9 | 6.06135 | [M+H]^+^ | Carveol | 135.118 | C_10_H_16_O |
| 10 | 6.395167 | [M+H]^+^ | Butyrolactone I | 425.0158 | C_24_H_24_O_7_ |
| 11 | 6.395167 | [M+H]^+^ | 2,6-Dimethoxyquinone | 186.0759 | C_8_H_8_O_4_ |
| 12 | 6.68665 | [M+H]^+^ | Limonene | 137.1329 | C_10_H_16_ |
| 13 | 7.146966 | [M+H]^+^ | Piperine | 570.8884 | C_17_H_19_NO_3_ |
| 14 | 7.364284 | [M+2H]^2+^ | Gelsenicine | 327.1725 | C_19_H_22_N_2_O_3_ |
| 15 | 7.8471 | [M+H]^+^ | Glycoursodeoxycholic acid | 488.2811 | C_26_H_43_NO_5_ |
| 16 | 8.667067 | [M+H]^+^ | Cynaropicrin | 369.1819 | C_19_H_22_O_6_ |
| 17 | 8.80005 | [M+2H]^2+^ | Octocrylene | 362.221 | C_24_H_27_NO_2_ |
| 18 | 8.806883 | [M+H]^+^ | α-Humulene | 243.1521 | C_15_H_24_ |
| 19 | 9.296534 | [M+2H]^2+^ | 5α-Cholestan-3β-Ol-6-one | 403.2599 | C_27_H_46_O_2_ |
| 20 | 10.35932 | [M+2H]^2+^ | Scalarin | 445.2887 | C_27_H_40_O_5_ |
| 21 | 10.73613 | [M+2H]^2+^ | Tetrahydrogambogic acid | 633.3375 | C_38_H_48_O_8_ |
| 22 | 11.39327 | [M+H]^+^ | p-Coumaric acid | 165.0535 | C_9_H_8_O_3_ |
| 23 | 11.39993 | [M+H]^+^ | Diallyl sulfide | 115.0543 | C_6_H_10_S |
| 24 | 11.39993 | [M+H]^+^ | Trans-cinnamaldehyde | 133.0683 | C_9_H_8_O |
| 25 | 12.6712 | [M+H]^+^ | Agnuside | 483.9656 | C_22_H_26_O_11_ |
| 26 | 15.13692 | [M+H]^+^ | Isoferulic acid | 217.0475 | C_10_H_10_O_4_ |
| 27 | 15.56657 | [M+H]^+^ | Isosafrole | 185.0594 | C_10_H_10_O_2_ |
| 28 | 15.7589 | [M+H]^+^ | 1-Naphthoic acid methyl ester | 187.0764 | C_12_H_10_O_2_ |
| 29 | 16.05672 | [M+H]^+^ | Ligustilide | 191.1055 | C_12_H_14_O_2_ |
| 29 | 14.9955 | [M−H]^−^ | Ligustilide | 189.0909 | C_12_H_14_O_2_ |
| 30 | 16.22703 | [M+H]^+^ | Dihydrojasmone | 167.1408 | C_11_H_18_O |
| 31 | 16.66802 | [M+H]^+^ | (E)-2-decylpent-2-enedioic acid | 293.1405 | C_15_H_26_O_4_ |
| 32 | 17.56865 | [M+H]^+^ | 2-(7-methoxy-2-oxochromen-8-yl)-3-methylbut-2-enal | 297.244 | C_15_H_14_O_4_ |
| 33 | 17.6128 | [M+H]^+^ | Thymoquinone | 187.0728 | C_10_H_12_O_2_ |
| 34 | 17.69863 | [M+H]^+^ | Dibutyl phthalate | 279.1611 | C_16_H_22_O_4_ |
| 35 | 18.07612 | [M+H]^+^ | Aurapten | 337.1381 | C_19_H_22_O_3_ |
| 36 | 18.11262 | [M+H]^+^ | 2-Methoxy-4-vinylphenol | 151.0726 | C_9_H_10_O_2_ |
| 37 | 18.12612 | [M+H]^+^ | 2-Hydroxyacetophenone | 137.0591 | C_8_H_8_O_2_ |
| 38 | 18.13945 | [M+H]^+^ | Cholestane | 316.1353 | C_27_H_48_ |
| 39 | 18.18645 | [M+H]^+^ | Bakuchiol | 257.1782 | C_18_H_24_O |
| 40 | 18.22145 | [M+H]^+^ | Clausarin | 381.2051 | C_24_H_28_O_4_ |
| 41 | 18.97475 | [M+H]^+^ | 5-Demethylnobiletin | 389.2685 | C_20_H_20_O_8_ |
| 42 | 18.97475 | [M+H]^+^ | Nerol | 177.128 | C_10_H_18_O |
| 43 | 18.97475 | [M+H]^+^ | 4-Hydroxyphenylethanol | 139.0768 | C_8_H_10_O_2_ |
| 44 | 19.0909 | [M+H]^+^ | Anethole | 171.0781 | C_10_H_12_O |
| 45 | 19.2864 | [M+H]^+^ | d-Limonene | 159.1175 | C_10_H_16_ |
| 46 | 19.45572 | [M+H]^+^ | Coumarin | 147.0424 | C_9_H_6_O_2_ |
| 47 | 19.85503 | [M+H]^+^ | Triptophenolide | 313.1799 | C_20_H_24_O_3_ |
| 48 | 21.50313 | [M+H]^+^ | Isopalmitic acid | 279.2294 | C_16_H_32_O_2_ |
| 49 | 22.78523 | [M+H]^+^ | Catechin tetramethylether | 347.2032 | C_19_H_22_O_6_ |
| 50 | 23.08572 | [M+H]^+^ | Dehydroabietic acid | 323.1982 | C_20_H_28_O_2_ |
| 51 | 24.85813 | [M+2H]^2+^ | Capsanthone-3,6-epoxide | 598.4071 | C_40_H_54_O_4_ |
| 52 | 25.8036 | [M+2H]^2+^ | Antheraxanthin | 584.3961 | C_40_H_56_O_3_ |
| 53 | 26.74688 | [M+H]^+^ | Oleic acid | 283.2633 | C_18_H_34_O_2_ |
| 54 | 15.34948 | [M−H]^−^ | 3,4-Dimethoxycinnamic acid | 207.066 | C_11_H_12_O_4_ |
| 55 | 15.75297 | [M−H]^−^ | Thymol | 149.0968 | C_10_H_14_O |
| 56 | 16.47677 | [M−H]^−^ | Gangaleoidin | 411.066 | C_18_H_14_C_l2_O_7_ |
| 57 | 16.52393 | [M−H]^−^ | Purpurogallin | 219.1394 | C_11_H_8_O_5_ |
| 58 | 22.21617 | [M−H]^−^ | 16-Hydroxyhexadecanoic acid | 271.2268 | C_16_H_32_O_3_ |
| 59 | 25.34568 | [M−H]^−^ | Sclareol | 307.2644 | C_20_H_36_O_2_ |

# 2 Result of GC–MS Based Qualitative Analysis of LCH injection

A total of 25 components were identified in LCH injection using GC–MS analysis and the retention time of Ligustilide was 27.792 min.

Figure S3. GC−MS chromatogram of LCH injection in negative ion mode

Table S2. Identified components from LCH injection by GC−MS

| **No.** | **RT(min)** | **Name** | **Peak area** | **Area percentage (%)** | **MW** | **Formula** |
| --- | --- | --- | --- | --- | --- | --- |
| 1 | 3.006 | Ethyl pentyl ether | 10367 | 0.16 | 116 | C_7_H_16_O |
| 2 | 3.15 | Toluene | 77109 | 1.19 | 92 | C_7_H_8_ |
| 3 | 3.314 | Butanoic acid | 16998 | 0.26 | 88 | C_4_H_8_O_2_ |
| 4 | 3.666 | 3-methoxy-1,2-Propanediol | 115241 | 1.78 | 106 | C_4_H_10_O_3_ |
| 5 | 4.811 | o-Xylene | 20977 | 0.32 | 106 | C_8_H_10_ |
| 6 | 5.264 | Cyclooctatetraene | 8647 | 0.13 | 104 | C_8_H_8_ |
| 7 | 8.64 | o-Cymene | 45644 | 0.71 | 134 | C_10_H_14_ |
| 8 | 13.245 | 4-Terpineol | 188865 | 2.92 | 154 | C_10_H_18_O |
| 9 | 13.387 | α,α,4-trimethyl- benzenemethanol | 28629 | 0.44 | 150 | \| C_10_H_14_O \| \| --- \| |
| 10 | 13.668 | *α*-Terpineol | 24226 | 0.37 | 154 | C_10_H_18_O |
| 11 | 16.845 | Cuprenenol | 50960 | 0.79 | 222 | C_15_H_26_O |
| 12 | 17.892 | 1,4-cyclohexadiene-1,2-Dicarboxylic anhydride | 71255 | 1.1 | 150 | C_8_H_6_O_3_ |
| 13 | 21.964 | *β*-Eudesmene/*α*-Guaiene | 39227 | 0.61 | 204/204 | C_15_H_24_/C_15_H_24_ |
| 14 | 22.212 | Butylated Hydroxytoluene | 479181 | 7.41 | 220 | C_15_H_24_O |
| 15 | 25.908 | 3-Butylphthalide | 481712 | 7.45 | 190 | C_12_H_14_O_2_ |
| 16 | 26.394 | Z-Butylidenephthalide | 488933 | 7.56 | 188 | C_12_H_12_O_2_ |
| 17 | 26.857 | 5-pentyl-1,3-Cyclohexadiene | 269547 | 4.17 | 150 | C_11_H_18_ |
| 18 | 27.47 | Senkyunolide A | 1197193 | 18.51 | 192 | C_12_H_16_O_2_ |
| 19 | 27.634 | trans-Sedanolide | 787898 | 12.18 | 194 | C_12_H_18_O_2_ |
| 20 | 27.792 | (E)-Ligustilide | 434420 | 6.71 | 190 | C_12_H_14_O_2_ |
| 21 | 32.55 | Dibutyl phthalate | 57826 | 0.89 | 278 | C_16_H_22_O_4_ |
| 22 | 32.658 | n-Hexadecanoic acid | 13081 | 0.2 | 256 | C_16_H_32_O_2_ |
| 23 | 32.968 | Senkyunolide N | 19191 | 0.3 | 226 | C_12_H_18_O_4_ |
| 24 | 33.738 | Senkyunolide H | 1369953 | 21.18 | 224 | C_12_H_16_O_4_ |
| 25 | 36.166 | trans-Elaidic acid | 172324 | 2.66 | 282 | C_18_H_34_O_2_ |
